# Carfilzomib relieves pancreatitis-initiated pancreatic ductal adenocarcinoma by inhibiting high-temperature requirement protein A1

**DOI:** 10.1038/s41420-024-01806-w

**Published:** 2024-01-29

**Authors:** Fangyue Guo, Xufeng Tao, Yu Wu, Deshi Dong, Yanna Zhu, Dong Shang, Hong Xiang

**Affiliations:** 1 Laboratory of Integrative Medicine, First Affiliated Hospital of Dalian Medical University, Dalian, 116011 China; 2 Institute (College) of Integrative Medicine, Dalian Medical University, Dalian, 116044 China; 3 Department of Pharmacy, First Affiliated Hospital of Dalian Medical University, Dalian, 116011 China; 4 Department of General Surgery, First Affiliated Hospital of Dalian Medical University, Dalian, 116011 China

**Keywords:** Cancer, Medical research

## Abstract

Pancreatitis is a crucial risk factor for pancreatic ductal adenocarcinoma (PDAC), and our previous study had proved high-temperature requirement protein A1 (HTRA1) exacerbates pancreatitis insult; however, the function and mechanism of HTRA1 in pancreatitis-initiated PDAC is still unclear. In the present paper, we clarified the expression of HTRA1 in PDAC using bioinformatics and immunohistochemistry of tissue chip, and found that HTRA1 is significantly upregulated in PDAC. Moreover, the proliferation, migration, invasion and adhesion of PANC-1 and SW1990 cells were promoted by overexpression of HTRA1, but inhibited by knockdown of HTRA1. Meanwhile, we found that HTRA1 arrested PANC-1 and SW1990 cells at G2/M phase. Mechanistically, HTRA1 interacted with CDK1 protein, and CDK1 inhibitor reversed the malignant phenotype of PANC-1 and pancreatitis-initiated PDAC activated by HTRA1 overexpression. Finally, we discovered a small molecule drug that can inhibit HTRA1, carfilzomib, which has been proven to inhibit the biological functions of tumor cells in vitro and intercept the progression of pancreatitis-initiated PDAC in vivo. In conclusion, the activation of HTRA1-CDK1 pathway promotes the malignant phenotype of tumor cells by blocking the cell cycle at the G2/M phase, thereby accelerating pancreatitis-initiated PDAC. Carfilzomib is an innovative candidate drug that can inhibit pancreatitis-initiated PDAC through targeted inhibition of HTRA1.

## Introduction

Pancreatic cancer (PC) is one of the most lethal gastrointestinal cancers that will become the 2nd leading cause of cancer-related mortality by 2030 [1]. Pancreatic ductal adenocarcinoma (PDAC) mainly originates from the pancreatic ductal epithelium and acinar cells, accounting for more than 90% of all PC cases [2]. Majority of PDAC patients are diagnosed at an advanced stage due to early asymptomatic or atypical symptoms, and not candidates for surgery [3]. The chemoradiotherapy of PDAC are constantly updated, however, the existing clinical options enhance survival by no more than a few months, resulting in a 5-year survival rate is only 12% [4]. Consequently, defining the mechanism that underlie the onset of PDAC is of great importance for early intervention.

PDAC is considered the most RAS-addicted of all cancers with a near 100% KRAS mutation frequency, and KRAS mutations have been reported in more than 95% of the precancerous pancreatic intraepithelial neoplasia (PanIN) [5]. Although KRAS mutation is a driver for PDAC, it alone is insufficient to promote tumorigenesis in absent of sustained environmental stress to achieve sufficient RAS activity [6]. Pancreatitis is an established risk factor for PDAC characterized by acinar cells damage and reprogramming [7]. Pancreatic acinar cells with high plasticity can transdifferentiate into a progenitor-like phenotype with ductal characteristics, termed acinar-to-ductal metaplasia (ADM) [8]. Acinar cells undergo ADM during pancreatitis, which is reversible once the injury is resolved. As a predominant form of atypical hyperplasia in pancreas, ADM exhibits a tumorigenic potential in the presence of oncogenic KRAS mutations [9]. However, the mechanisms underlying initiation of pancreatitis-initiated PDAC remain unresolved.

High-temperature requirement protein A1 (HTRA1) is the first discovered member of the human HTRA serine protease family. As a secreted protein, HTRA1 is related to the degradation of extracellular matrix [10]. In addition to serving as a tumor marker and/or prognostic factor, HTRA1 is also connected to tumorigenesis via regulating tumor cell proliferation, migration, apoptosis and differentiation [11, 12]. Our previous research is expected to identify another HTRA1-mediated mechanism for pancreatitis-initiated PDAC initiation, in which, HTRA1 is significantly up-regulated during pancreatitis [13]. Here, we attempted to investigate the molecular mechanism by which highly expressed HTRA1 promotes the malignant phenotype of pancreatic tumor cells, as well as PanIN and PDAC progression in pancreatic tissues, and further provided a candidate drug for impeding pancreatitis-initiated PDAC through targeted inhibition of HTRA1.

## Result

### HTRA1 was up-regulated in PDAC

**Fig. 1:**
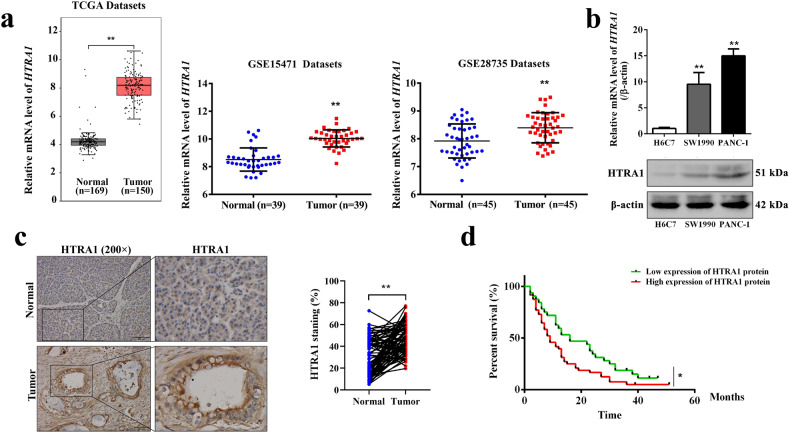
HTRA1 was up-regulated in PDAC. **a** *HTRA1* expression is significantly elevated in PDAC compared to normal, in the TGCA database, GSE15471, and GSE28735. **b** HTRA1 mRNA and protein expression levels were upregulated in SW1990 and PANC-1 cells compared with H6C7 cells. **c** HTRA1 protein expression was increased in tumor tissues compared with normal pancreatic tissues (magnification, ×200). **d** Survival was shorter in patients with high HTRA1 expression (*n* = 80). Data are presented as the mean ± SD. *p < 0.05, **p < 0.01 vs. Normal or H6C7.

To clarify the expression of HTRA1 in PDAC, we first queried a data set composed of 169 paracancerous tissues and 150 PDAC tissues from TGCA database and GTEx database through bioinformatics analysis. As shown in Fig. 1a, the relative mRNA expressions of *HTRA1* were significantly increased in PDAC compared with the normal. To further verify this result, we searched and downloaded two PDAC datasets (GSE15471 and GSE28735) containing *HTRA1*expression data, and obtained results consistent with TCGA data (Fig. 1a). Subsequently, we validated the expression of HTRA1 in PDAC through in vivo and in vitro experiments, respectively. In vitro, both the gene and protein expressions of HTRA1 in PDAC cell lines, PANC-1 cells and SW1990 cells, were significantly higher than that of H6C7 cells (Fig. 1b). In vivo, immunohistochemistry of human tissue microarray of PDAC confirmed that the upregulation of HTRA1 was observed in PDAC tissues (Fig. 1c). According to the survival data of 80 PDAC patients, we found that the percent survival of patients with high expression of HTRA1 protein had shorter survival time and higher risk of prognosis than patients whose HTRA1 protein expression were low (Fig. 1d). These results suggest that elevated HTRA1 levels may be associated with the occurrence, development, and prognosis of PDAC.

### Overexpression of *HTRA1* promoted the biological functions of PANC-1 and SW1990 cell

**Fig. 2:**
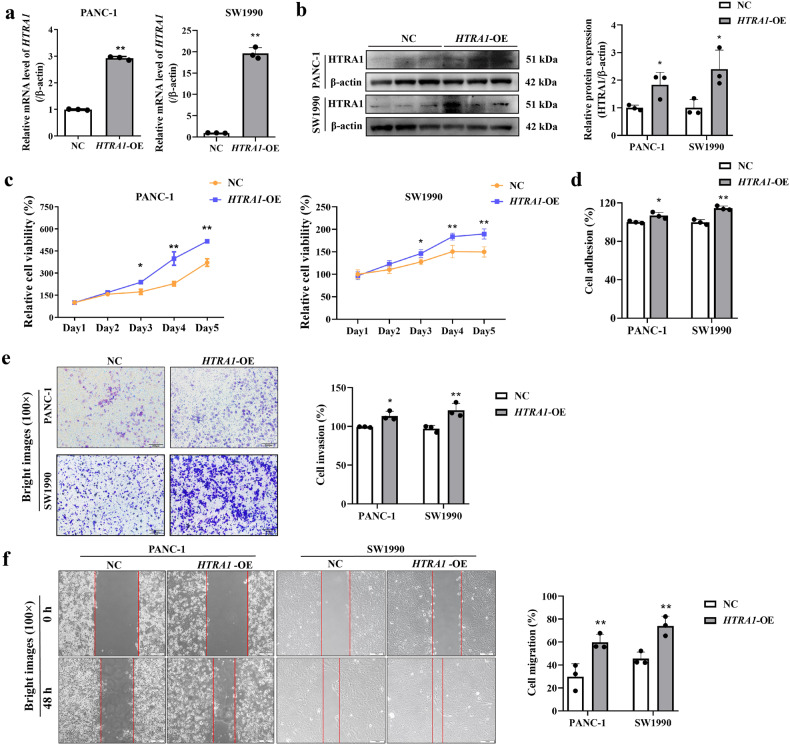
Overexpression of HTRA1 promoted the biological functions of PANC-1 and SW1990 cells. **a** *HTRA1* mRNA levels were upregulated in *HTRA1*-overexpressing PANC-1 and SW1990 cells (*n* = 3). **b** Protein expression levels of HTRA1 were upregulated in *HTRA1*-overexpressing PANC-1 and SW1990 cell (*n* = 3). **c** Cell viability of PANC-1 and SW1990 cells were notably upregulated in *HTRA1*-overexpressing PANC-1 and SW1990 cells at day 3, 4 and 5 (*n* = 6). **d**, **e** *HTRA1*-overexpressing significantly increased the cell adhesion and invasion (*n* = 3). **f** Representative images shown that *HTRA1*-overexpressing enhanced migration capacities of PANC-1 and SW1990 cells. (magnification, ×100). Data are presented as the mean ± SD. *p < 0.05, **p < 0.01 vs. NC.

As shown in Fig. 2a, b, HTRA1 mRNA level and protein level was significantly upregulated in PANC-1 and SW1990 cells after transfection with the lentivirus overexpressing *HTRA1* (*HTRA1-*OE). The effect of HTRA1 on cell proliferation was detected by CCK-8 method, and the results showed that the cell viability of PANC-1 and SW1990 cells were notably upregulated after *HTRA1*-overexpressing at day 3, 4 and 5, suggested that overexpression of *HTRA1* promoted the proliferation of PANC-1 and SW1990 cells (Fig. 2c). It can be found that cell adhesion abilities were increased after overexpressing *HTRA1* in PANC-1 and SW1990 cells (Fig. 2d). Similarly, Transwell experiment found that the number of invasive cells was significantly increased with the upregulation of *HTRA1* in PANC-1 and SW1990 cells (Fig. 2e). In addition, overexpression of *HTRA1* increased the migration ability of PANC-1 and SW1990 cells through cell scratch experiments (Fig. 2f).

### Silencing of *HTRA1* inhibited the malignant phenotype of PANC-1 and SW1990 cell but promoted apoptosis and induce cell cycle arrest at the G2/M phase

**Fig. 3:**
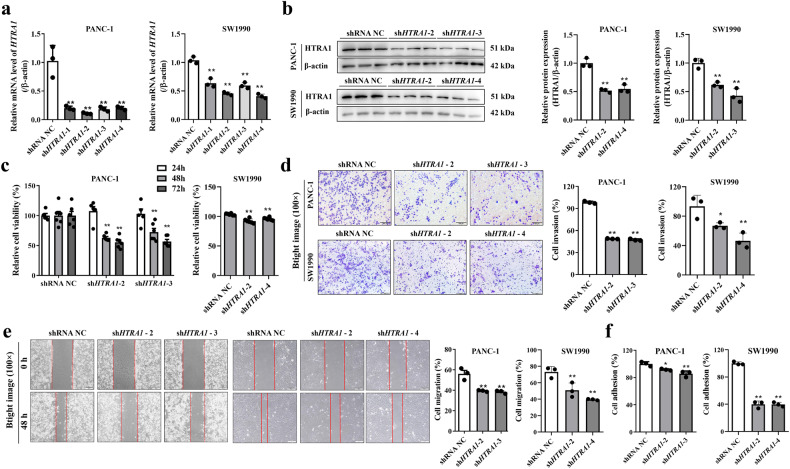
Silencing of *HTRA1* inhibited the malignant phenotype of PANC-1 and SW1990 cells. **a**, **b** sh*HTRA1*-2 and -3(or -4) significantly downregulated *HTRA1* mRNA (*n* = 3) and protein expressions (*n* = 3) in PANC-1 and SW1990 cells. **c** Knockdown of *HTRA1* significantly increased the cell viability of PANC-1 cells at 48 and 72 h and significantly increased the cell viability of SW1990 cells at 48 h (*n* = 6). **d**, **e** Representative images and statistics of migrated or invaded cells shown that knockdown of *HTRA1* significantly weakened migration and invasion capacities of PANC-1 and SW1990 cell. (*n* = 3) (magnification, ×100). **f** Knockdown of HTRA1 significantly inhibited the adhesion of PANC-1 and SW1990 cells (*n* = 3). Data are presented as the mean ± SD. *p < 0.05, **p < 0.01 vs. shRNA NC.

**Fig. 4:**
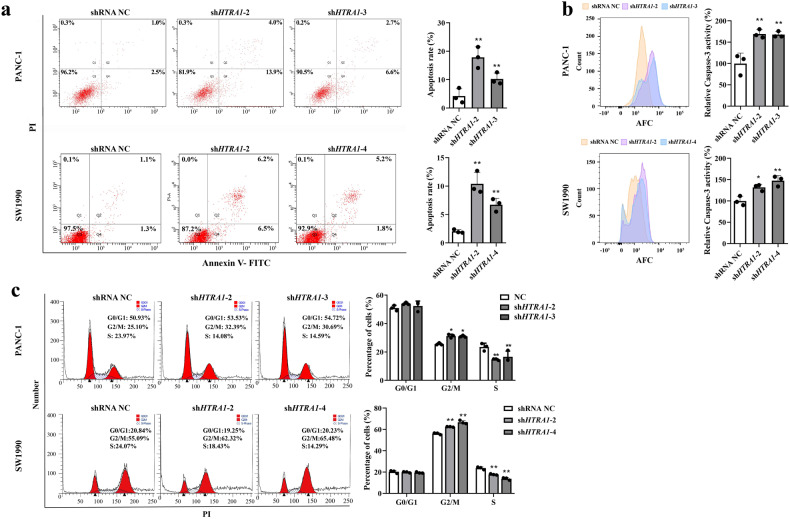
Silencing of *HTRA1* promoted apoptosis and caspase-3 activity and induce cell cycle arrest at the G2/M phase. **a**, **b** Knockdown of *HTRA1* notably increased apoptosis and caspase-3 activity of PANC-1 and SW1990 cells (*n* = 3). **c** Knockdown of *HTRA1* showed cell cycle arrested at the G2/M phase in PANC-1 and SW1990 cells (*n* = 3). Data are presented as the mean ± SD. *p < 0.05, **p < 0.01 vs. shRNA NC.

To reverse verify the function of HTRA1 in PDAC progression, we knocked down *HTRA1* in PANC-1 and SW1990 cells by transfection of shRNA plasmids. Four types of sh*HTRA1* were transfected into cells, sh*HTRA1*-2 and -3 had the best knockdown efficiency in PANC-1 cells, and sh*HTRA1*-2 and -4 had the best knockdown efficiency in SW1990 cells. (Fig. 3a, b). As shown in Fig. 3c, knockdown of *HTRA1* expression significantly inhibited the proliferation ability of PANC-1 cells at 48 and 72 h, but no impact at 24 h, similarly, the proliferation ability of SW1990 cells was also inhibited at 48 h. Contrary to the results of overexpression of *HTRA1*, after down-regulation of *HTRA1* expression in PANC-1 and SW1990 cells, cell migration ability, number of invasive cells and cell adhesion activity were significantly reduced (Fig. 3d–f). In addition, flow cytometry showed that the proportion of total cells in Q2 and Q4 in the sh*HTRA1* groups and caspase-3 activity were significantly higher than that in the NC group (Fig. 4a, b). Further, we detected the cell cycle after knockdown of *HTRA1* in PANC-1 and SW1990 cells, the percentage of cells had none significant changes in G0/G1 phase, but increased significantly in G2/M phase, and decreased notably in S phase, suggesting that PANC-1 and SW1990 cells were arrested at the G2/M phase upon *HTRA1* knockdown (Fig. 4c). These results confirmed that *HTRA1* silencing inhibited the proliferation, migration, invasion and adhesion of PANC-1 and SW1990 cells, and promoted apoptosis, and mainly promotes G2/M phase in the cell cycle.

### HTRA1 interacted with CDK1 protein

**Fig. 5:**
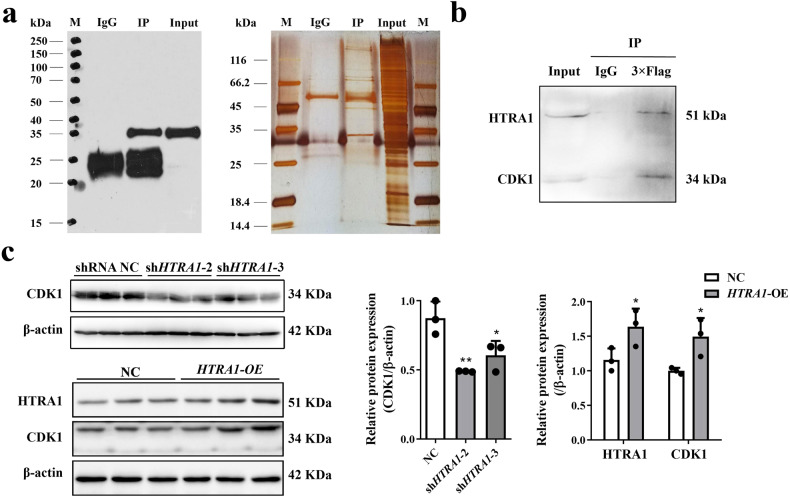
HTRA1 protein interacted with CDK1 protein. **a** Silver staining and western blot results of HTRA1-IP experiment. **b** Co-IP experiment results further indicated that HTRA1 protein can pull down the CDK1 protein. **c** Protein expression levels of CDK1 were downregulated after knocking down *HTRA1* in PANC-1 cells and upregulated in *HTRA1*-overexpressing PANC-1 cells (*n* = 3). Data are presented as the mean ± SD. *p < 0.05, **p < 0.01 vs. shRNA NC or NC.

In order to explore the potential mechanism of HTRA1 affecting the biological functions of PDAC cells, we performed HTRA1-IP experiment. The results of western blot found that anti-HTRA1 can specifically bind to the target protein, and silver staining indicated that HTRA1 pulled down several different proteins compared to IgG without specifically binding to IgG (Fig. 5a). LC-MS/MS analysis recognized 293 of unique pull-down proteins in the HTRA1 group, which has been shown in Supplementary Table S1. Among them Cyclin dependent kinase 1 (CDK1) was found to play an important role in G2/M phase of the cell cycle. CDK1 is a highly conserved Ser/Thr protein kinase, as a key protein that drives mitotic onset in all eukaryotes [14]. The Co-IP results further verified that HTRA1 protein can pull down CDK1 protein, which pointed out the interaction between HTRA1 protein and CDK1 protein (Fig. 5b). As shown in Fig. 5c, the protein expression levels of CDK1 downregulated by both sh*HTRA1*-2 and -3, but up-regulated by overexpression of *HTRA1* in PANC-1 cells.

### HTRA1 promoted cell adhesion, migration and invasion by targeting CDK1-mediated cell cycle

**Fig. 6:**
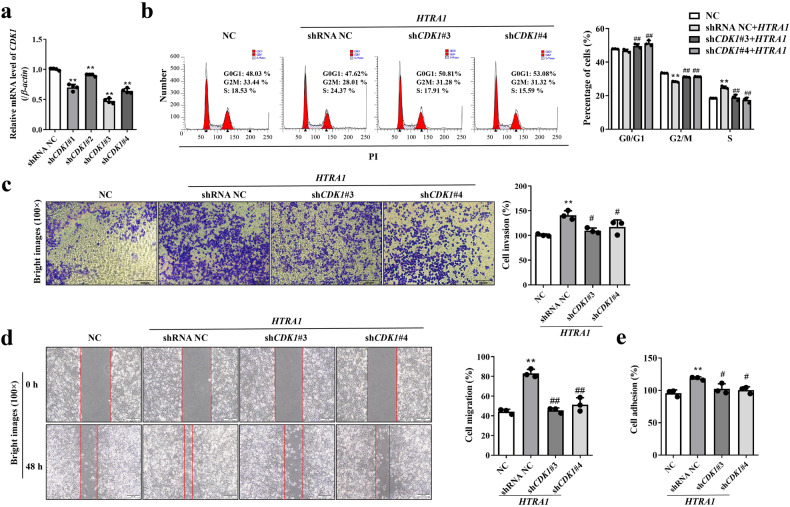
HTRA1 promoted cell adhesion, migration and invasion by targeting CDK1-mediated cell cycle. **a** sh*CDK1*s significantly downregulated CDK1 mRNA level compared to shRNA NC group (*n* = 3). **b** Knockdown of *CDK1* reversed cell cycle promote at G2/M phase in *HTRA1*-overexpressing PANC-1 cells (*n* = 3). **c**–**e** Knockdown of *CDK1* weakened migration, invasion and adhesion capacities in *HTRA1*-overexpressing PANC-1 cells (*n* = 3) (magnification, ×100). Data are presented as the mean ± SD. **p < 0.01 vs. NC; ^#^*p* < 0.05, ^##^*p* < 0.01 vs. shRNA NC.

To validate whether CDK1 is a downstream interacting protein of HTRA1, we constructed four shRNA plasmids, sh*CDK1*-#1, -#2, -#3 and -#4, and found that the knockdown efficiency of sh*CDK1*-#3 and -#4 can reach 50–60% (Fig. 6a). Therefore, we transfected sh*CDK1*-#3 and -#4 into *HTRA1*-overexpressing PANC-1 cell line, and then detected the cell cycle of these cells upon downregulation of *CDK1* expression. As shown in Fig. 6b, after overexpression of *HTRA1*, PANC-1 cell cycle was decreased at G2/M phase, while knockdown of *CDK1* could significantly decreased the percentage of cells in G0/G1 phase and S phase, but increased the percentage of cells in G2/M phase. These results hinted that *CDK1* depletion reversed cell cycle promote at G2M phase causing by overexpression of *HTRA1*. The biological function of cells is regulated by CDK1 during the cell cycle [15]. Therefore, we detected the adhesive, migratory, and invasive capabilities of PANC-1 cells overexpressing *HTRA1* upon downregulation of *CDK1*. The results shown that compared with the NC group (with shRNA NC), the migration and invasion of PANC-1 cells overexpressing *HTRA1* was significantly increased, but knockdown of *CDK1* could reverse this progress (Fig. 6c, d). Moreover, inhibition of *CDK1* with sh*CDK1*s transfection significantly reduced the adhesion of PANC-1 cells induced by *HTRA1* overexpression (Fig. 6e). These results proved that elevated HTRA1 can promote the adhesion, migration and invasion of PANC-1 cells via interacting with CDK1-mediated cell cycle.

### HTRA1 accelerated pancreatitis-initiated PDAC in KC mice

**Fig. 7:**
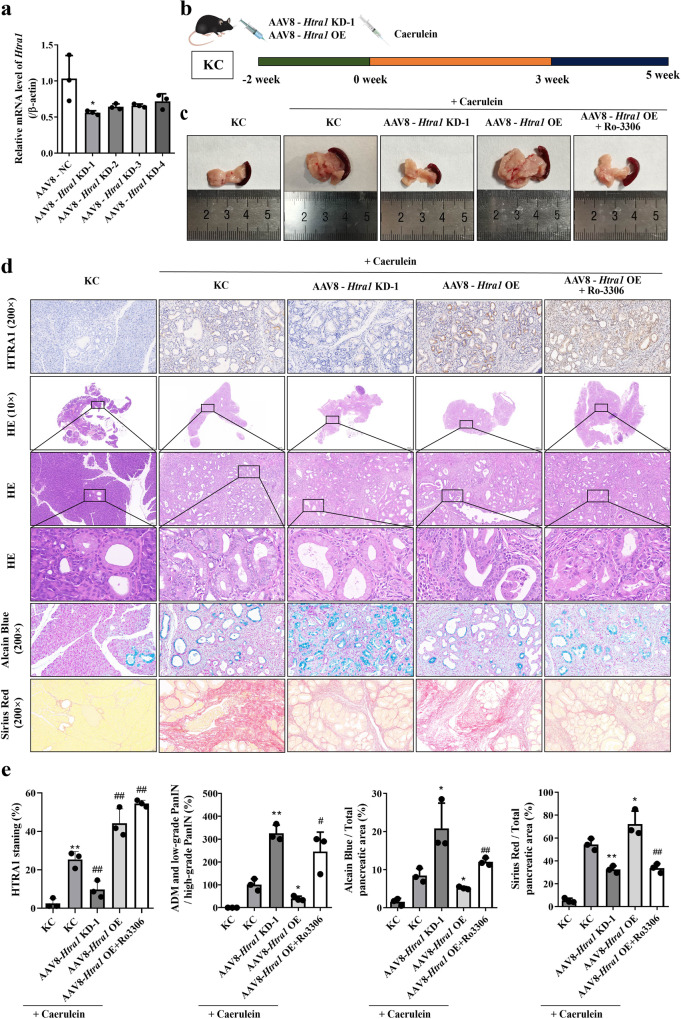
HTRA1 accelerated pancreatitis-initiated PDAC in KC mice. **a** AAV8- *Htra1* KD-1 significantly downregulated *Htra1* mRNA in PANC02 cell (*n* = 3). **b** Pancreatitis was induced in KC mice 2 weeks after the AAV virus was injected into the pancreas, and the mice were killed at a specified time point after the last injection. **c** CDK1 inhibitor reversed an increase of pancreatic volume and mass induced by *HTRA1* overexpression in KC mice expose to cerulein. **d** HTRA1 staning show that AAV8-*Htra1* KD-1 downregulated HTRA1 level and AAV8-*Htra1* OE upregulated HTRA1 level in KC mice pancreas. HE, Alcian blue, and Sirius red staining shown that cerulein induced ADM in KC mice progression to high grade of PanIN, and collagen deposition with the upregulation of *Htra1*, which can be reversed by a CDK1 inhibitor, Ro3306. (magnification, ×100). **e** Statistics of pancreatic lesions by HE, Alcian blue, and Sirius red staining (*n* = 3). Data are presented as the mean ± SD. *p < 0.05, **p < 0.01 vs. KC + caerulein; ^#^*p* < 0.05, ^##^*p* < 0.01 vs. AAV8-*Htra1* OE + caerulein.

Due to the significance exhibited by HTRA1 in vitro, we constructed four kinds of AAV8-*Htra1* KDs for gene silencing of *Htra1* in vivo. It was observed that AAV8-*Htra1* KD-1 had the best suppression efficiency that can be selected for subsequent in vivo experiments (Fig. 7a). As a replacement for transplant models, genetically engineered mouse (GEM)-based PDAC models provide a better option for preclinical therapeutic evaluation. KC mice conditionally express endogenous mutant KRAS alleles in pancreatic cells and develop pancreatic tumors with pathological, physiological, and molecular features similar to human PDAC [16, 17]. Thus, we used KC mice model to recapitulate PDAC initiation and investigated the effects of HTRA1 on pancreatic tumors in vivo. Intraperitoneal injection of cerulein is a classic method for inducing pancreatitis, which has been proven to be an independent risk factor for PDAC [18]. The timeline of different processing methods has been shown in Fig. 7b. As shown in Fig. 7c, the volume and mass of the pancreas collected from KC mice treated with cerulein were higher than that of KC mice without other treatments, as a result of the occurrence and development of pancreatic tumors. Compared to the group treated with only caerulein, pancreatic volume and mass exhibited a significant decrease after inhibiting *Htra1* expression (Caerulein + AAV8-*Htra1* KD-1 group), but showed a significant increase in KC mice overexpressing *Htra1* (Caerulein + *Htra1* OE group). Interestingly, treatment with a CDK1 inhibitor (Caerulein + *Htra1* OE + Ro-3306) reversed an increase of pancreatic volume and mass induced by *Htra1* overexpression in KC mice expose to cerulein. Morever, we proved by immunohistochemical HTRA1 staining that compared with Caerulein + KC group, the expression of HTRA1 protein was significantly down-regulated in Caerulein + AAV8-*Htra1* KD-1 group, while it was significantly up-regulated in Caerulein + *Htra1* OE group and Caerulein + *Htra1* OE + Ro3306 groups (Fig. 7d, e). To verify whether HTRA1 accelerates PDAC in KC mice, we performed H&E staining on the pancreatic tissue of KC mice in different treatment groups to evaluate the pancreatic injury and PanIN lesions. Pathology of pancreatic tissues showed that a few of ADM spontaneously formed in pancreatic tissue of KC mice without other treatments, while cerulein stimulation accelerated pancreatic injury, ADM and even low-grade PanIN in KC mice. This progression can be reduced by suppression of *Htra1*. It is vigilance that cerulein induced pancreatic ADM in KC mice progression to high grade of PanIN lesions, and even partial progression to PDAC with the upregulation of *Htra1*, but pancreatitis-initiated PDAC after *Htra1* overexpression can be reversed by CDK1 inhibitor (Fig. 7d, e).

In this study, we used Alcian Blue staining to evaluate the severity of pancreatic lesions and PanIN in each group of KC mice. As shown in Fig. 7d, e, only a small amount of Alcian Blue positive staining (bule area) in pancreatic tissue of KC mice. After treatment with cerulein, KC mice showed significant pancreatic lesions, and the number of Alcian Blue positive staining was significantly increased, which can be reversed by silencing *Htra1*. Furthermore, KC mice with high-expression of *Htra1* had most high-grade PanIN in pancreatic tissue, but the number of high-grade PanIN lesions were reduced by CDK1 inhibition. Here, we used Sirius Red staining to determine collagen production in pancreatic tissue of KC mice in different treatment groups. The results shown that a large amount of collagen fibers was deposited in the pancreatic tissue of KC mice stimulated by cerulein, mainly manifested as an increase in the Sirius red positive staining (red area) compared to KC mice. *Htra1* deficiency resulted in reduced collagen deposition, while high-expression of *Htra1* increased collagen deposition. However, the pancreas of KC mice overexpressing *Htra1* showed a significant reduction in collagen deposition after treatment with CDK1 inhibitor (Fig. 7d, e). These results indicated that KC mice alone is not enough to develop into PDAC due to mild pancreatic lesions, however, the pancreatic tissues of KC mice progressed to high-level PanIN lesions and even PDAC after being stimulated with cerulein and *Htra1* overexpression. Therefore, the upregulation of *Htra1* in KC mice can accelerate pancreatitis-initiated PDAC.

### Carfilzomib has an inhibitory effect on the malignant phenotype of PDAC cells

**Fig. 8:**
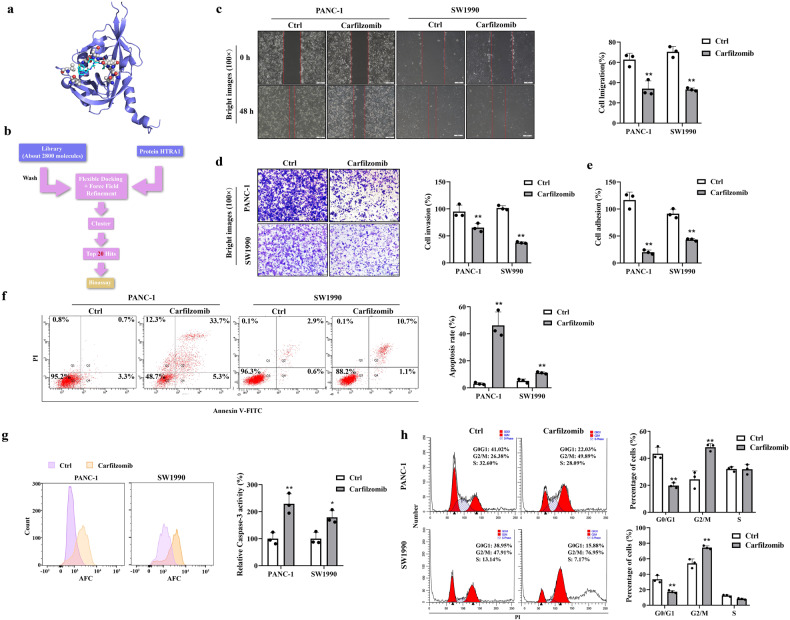
Small molecule drug that can inhibit HTRA1 decreased the malignant phenotype of PANC-1 cells. **a** The structure of HTRA1 was download from RCSB PDB Data Bank with PDB ID of 3NZI. **b** Flowchart of SBVS on small molecules. **c**, **d** Representative images and statistics shown that carfilzomib notably weakened migration and invasion of PANC-1 and SW1990 cells (*n* = 3). (magnification, ×100). **e** Carfilzomib notably weakened adhesion capacities of PANC-1 and SW1990 cells (*n* = 3). **f**, **g** Carfilzomib notably increased apoptosis and caspase-3 activity of PANC-1 and SW1990 cells (*n* = 3). **h** Carfilzomib arrested cell cycle at the G2/M phase in PANC-1 and SW1990 cells (*n* = 3). Data are presented as the mean ± SD. *p < 0.05, **p < 0.01 vs. Ctrl.

In order to find candidate drugs targeting HTRA1 for the treatment of pancreatitis-initiated PDAC, we first determine the binding pocket of HTRA1 by analyzing its 3D structure (Fig. 8a). Then, we screened small molecule inhibitors that interacts with HTRA1 from approved drug molecules at Drugbank by VS, and the flow of VS was shown in Fig. 8b. According to the binding mode and docking score of small molecule inhibitor and HTRA1, the top 20 of HTRA1 inhibitors were listed in Supplementary Fig. S1. Considering the effectiveness of the drug and the convenience of medication administration, we ultimately focus on four compounds suitable for injection therapy from the Top 10 compounds for experimental verification. The cytotoxicity of selected four compounds (pipecuronium bromide, succinylcholine chloride, carfilzomib and choline chloride) were verified on PANC-1 cells, and the results indicated that only carfilzomab had cytotoxicity within the selectable range of dosing concentration. Morever, in SW1990 cells, carfilzomab was equally cytotoxic, so we finally choose carfilzomab for in vitro validation (Supplementary Fig. S2a, b). As shown in Fig. 8c, d, we found that PANC-1 and SW1990 cells treated with 0.001 μM of carfilzomib had lower migration and invasion than the control. Moreover, 0.01 μM of carfilzomib could promote apoptosis and caspase-3 activity of PANC-1 and SW1990 cells but inhibit their adhesion (Fig. 8e–g). The evaluation of biological function of PANC-1 and SW1990 cells found that carfilzomib at 0.01 μM could significantly reduce the percentage of cells in G0/G1 phase and increase the percentage of cells in G2/M phase, but have no significant effect on S phase (Fig. 8h). The results suggested that carfilzomib can block PANC-1 cells at the G2/M phase of the cell cycle. Therefore, these results confirmed that carfilzomib has an inhibitory effect on the malignant phenotype of PDAC cells.

**Fig. 9:**
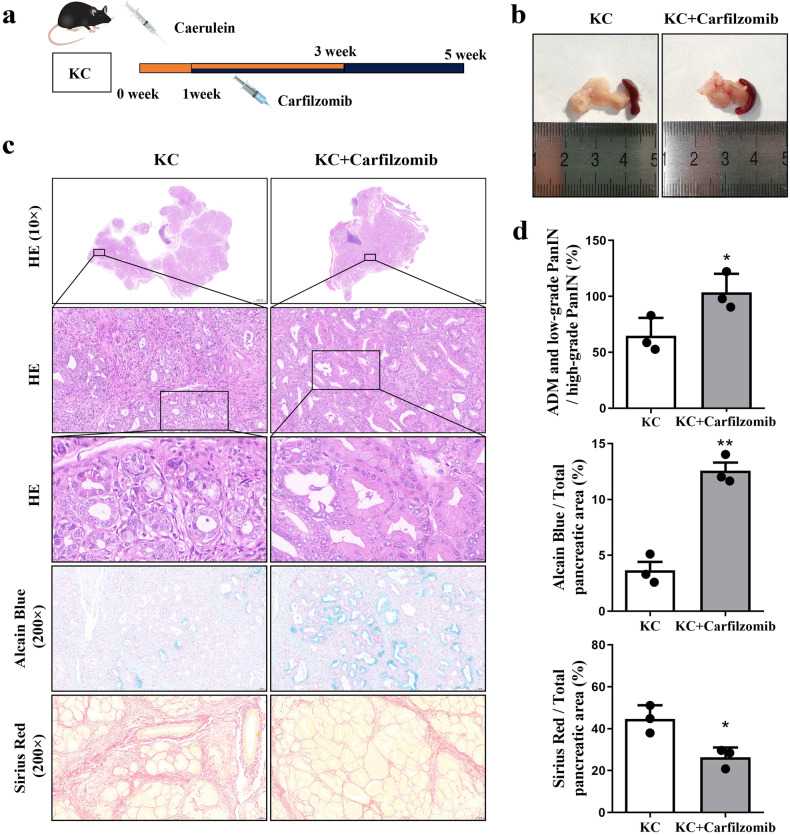
Carfilzomib inhibited pancreatitis progression to PDAC in KC mice. **a** KC mice were treated with carfilzomib at 1 week after induced pancreatitis and sacrificed at 5 weeks. **b** Gross pancreas morphology shown a reduction in tumor size following carfilzomib treatment in KC mice. **c** HE, Alcian blue and Sirius red staining revealed a decrease in high-grade PanIN lesions, but an increase in ADM and low-grade PanIN lesions in the pancreas of KC mice post carfilzomib intervention. (magnification, ×100). **d** Statistics of pancreatic lesions by HE, Alcian blue, and Sirius red staining. Data are presented as the mean ± SD. *p < 0.05, **p < 0.01 vs. KC.

In order to further investigate the effect of carfilzomib on PDAC, we conducted in vivo pharmacological experiments. After administering cerulein for 1 week, carfilzomib was intravenously injected into the tail vein at a dose of 5 mg/kg/week for five consecutive weeks (Fig. 9a). Observations of the external appearance of KC mice pancreas showed a reduction in tumor size following carfilzomib treatment (Fig. 9b). HE and Alcian blue staining revealed a decrease in high-grade PanIN lesions, but an increase in ADM and low-grade PanIN lesions in the pancreas of KC mice post carfilzomib intervention compared with the untreated KC mice (Fig. 9c, d). Additionally, Sirius Red staining observed the collagen deposition in pancreatic tissues of KC mice can be reduced by carfilzomib intervention (Fig. 9c, d). These results indicated that carfilzomib may effectively impede the progression of pancreatitis-initiated PDAC by suppressing HTRA1, which is promising to sever as an emerging targeted drug for PDAC therapy.

## Discussion

A long-established clinical observation demonstrated that inflammation is a major risk factor for PDAC. When pancreatitis occurs, inflammatory microenvironment accelerate tumor development with the activation of KRAS mutations [19]. Collectively, targeted blockade of sustained inflammatory stimuli is important for effectively preventing KRAS mutations induced-PDAC. The mutation of HTRA1 has been implicated in a plethora of disorders and this has also led to its growing interest as a drug target for future therapy [10]. The majority of cancer-related HTRA1 research hypothesized it as a tumor suppressor, such as, ovarian cancer, endometrial cancer, hepatocellular carcinoma, and melanoma [20–23]. However, there are also some different voices that current research on HTRA1 does not conclusively support its role as a tumor suppressor. The main contradiction lies in often the results were incomparable due to the different rating scales and study design [24]. Our previous research confirmed the high expression of HTRA1 in pancreatitis [13], which may open up new ideas for the effects and mechanisms of HTRA1 in pancreatitis-induced PDAC.

In this study, we firstly verified that the relative mRNA expressions of *HTRA1* in tumor tissues of PDAC patients was higher than that in paracancerous tissues through the existing database. In vitro and in vivo experiments, we have also confirmed the high expression of HTRA1 in PDAC, which was associated with a shorter survival time of PDAC patients. In order to further validate the role of HTRA1 in PDAC initiation, we overexpressed or knocked down *HTRA1* in PANC-1 and SW1990 cells with the lentivirus overexpressing *HTRA1* and sh*HTRA1*, respectively. The results shown that overexpression of *HTRA1* promoted the proliferation, migration, and adhesion of PANC-1 and SW1990 cell, providing further evidence for suggesting that HTRA1 servers as a dual role in cancers [25]. However, knockdown of *HTRA1* decreased the malignant phenotypes of PANC-1 and SW1990 cell, but increased apoptosis and caspase-3 activity. Caspase-3 activity plays an irreplaceable role in apoptosis; upon activation, caspase-3 hydrolyzates proteins, leading to DNA damage and inducing apoptosis [26–29]. In addition, knockdown of *HTRA1* could affect the cell cycle and arrest PANC-1 and SW1990 in G2/M phase.

To reveal how high expression of HTRA1 acts on PDAC progression, we performed IP and LC-MS/MS analysis, from the results, we found that CDK1, a protein highly associated with the cell cycle, interacted with HTRA1, has never been disclosed before. In addition, knockdown and overexpression of *HTRA1* correspondingly downregulated and upregulated CDK1. CDK1, one of the cell cycle master kinases, has critical regulatory roles in the cell cycle through the G2/M phase transition and reversion of DNA damage sensitivity [30]. Failure to accurately control CDK1 activity has been linked to the loss of cell cycle control, which lies at the heart of tumor growth [31]. The cell cycle machinery also regulates cell adhesion in manners recently shown driven mainly by CDK1 [32]. Here, the functions of HTRA1 in PDAC is implemented in a CDK1 dependent manner. Overexpression of *HTRA1* promoted malignant phenotype of pancreatic tumor cells in vitro and pancreatitis-induced PDAC in KC mice, however, inhibiting HTRA1 blocked cell cycle at the G2/M phase, thereby inhibiting the malignant phenotype of tumor cells and promoting apoptosis in vitro, and inhibited the progression of PDAC in KC mice. Therefore, HTRA1 may become a critical target for inhibiting the progression of pancreatitis-induced PDAC.

Traditional chemotherapy drugs have limited efficacy for PDAC patients, and targeted drugs also have been difficult. Erlotinib is the only precise therapeutic agent approved for PDAC, which only slightly prolongs survival [33]. Therefore, it is necessary to explore new targeted drugs for future treatment of PDAC. Drug repurposing offers us the possibility of better choices by utilizing existing drugs that have already been approved for treating one disease and reapplying them to treat other diseases. Drug repurposing can save a significant amount of time and resources, as the safety and pharmacokinetic properties of these drugs are already well-established. Additionally, it allows us to gain a deeper understanding of the mechanisms of action of these drugs and enables us to select more suitable medications to improve therapeutic outcomes for patients [34, 35]. Due to the importance of HTRA1 in pancreatitis-initiated PDAC, we employed DrugBank’s virtual screening to identify small molecule drug that can inhibit HTRA1 among the approved drug molecules. The results of cytotoxicity showed that only carfilzomab had cytotoxicity within the selectable range of dosing concentration.

Carfilzomib, a tetrapeptide epoxyketone, has been approved by American food and drug administration (FDA) in 2012 for treating patients with relapsed/refractory multiple myeloma. Carfilzomib blocks the degradation of dysfunctional proteins by irreversibly inhibiting the proteasome in its ChT-L site [36]. In addition, carfilzomib combined with paclitaxel, ganetespib, or HDAC class IIa selective inhibitor TMP269 have an effective therapy against PC [37–39]. Here, we first reported carfilzomib as a promising small molecule drug that can inhibit HTRA1. After a series of in vitro and in vivo experiments, we have finally confirmed that carfilzomib significantly inhibited the malignant phenotype of PDAC cells, and the progression of pancreatitis-initiated PDAC by suppressing HTRA1, which is promising to sever as an emerging targeted drug for PDAC therapy.

## Conclusions

HTRA1 is high expressed in PDAC, and severs as a critical activator for tumor cells malignant phenotype and pancreatitis-initiated PDAC progression by targeting CDK1-mediated cell cycle. Here, we highlighted the potential for inhibiting HTRA1 as a mean to impede pancreatitis-initiated PDAC, and further provided a newly discovered a small molecule drug that can inhibit HTRA1, carfilzomib, as candidate drug for PDAC therapy.

## Materials and methods

### Animals

Adeno-associated viruses (AAV) of serotype 8 for *Htra1* overexpression (AAV8-*Htra1* OE) and AAV8 *Htra1* mediated gene silencing (AAV8-*Htra1* KDs) were produced by GenePharma (Shanghai, China). Firstly, we validated the gene silencing efficiency of AAV8-*Htra1* KDs in PANC02 cells. Next, LSL-Kras(G12D/+); Pdx1-Cre (KC) mice (male, 25–30 g) were purchased from Cyagen Biosciences Inc. (Guangzhou, China). All mice were maintained under specific pathogen-free conditions at Cyagen Model Biological Research Center (Taicang) Co., Ltd. All experiments were performed in strict accordance with the People’s Republic of China Legislation regarding the Use and Care of Laboratory Animals. In vivo, mice were anesthetized using isoflurane inhalation, and then an incision in the left abdomen was made to expose the pancreas. About 100 μl of AAV8 vectors at a final dose of 2 × 10^11^ genome copies was injected into the splenic lobe of the pancreas. Successful delivery was considered by a localized swelling of pancreas at the site of injection. Two weeks after AVV8 injection, caerulein was administered as intraperitoneal injection 4 times a day (50 μg/kg), 3 days per week duration of 3 weeks. The mice were sacrificed 2 weeks after CP model establishment for pancreas collection.

### Cell culture

Human PDAC lines PANC-1, SW1990, human pancreatic duct cell line HPDE6-C7 (H6C7), and mouse PC line Panc02 were purchased from American Type Culture Collection (ATCC, VA, USA). Cells were maintained in Dulbecco’s modified Eagle’s medium (DMEM) with high glucose supplemented and 10% fetal bovine serum (FBS) in a humidified atmosphere of 5% CO_2_ at 37 °C. All cell lines by Short Tandem Repeat (STR) identification and detection of mycoplasma on a regular basis.

### Statistical analysis

Data were analyzed using GraphPad Prism 8.0 software (GraphPad; CA, USA) and expressed as the mean ± SD. Statistical analysis was determined using Student’s *t* test, one-way ANOVA or two-way ANOVA. *p* values of <0.05 or <0.01 were considered statistically significant.

### Supplementary information

Supplementary

Supplementary

## Data Availability

All data generated during this study leading to the findings presented here are included in this published article and its Supplementary Data files. All data are available from the corresponding author upon reasonable request.
