# Supplementary material for: Carfilzomib relieves pancreatitis-initiated pancreatic ductal adenocarcinoma by inhibiting high-temperature requirement protein A1
Source: Cell Death Discov. 2024 Jan 29;10:58. doi: 10.1038/s41420-024-01806-w (PMC10825157; doi:10.1038/s41420-024-01806-w)
Supplement: Supplementary file 1 — Supplementary [file 41420_2024_1806_MOESM1_ESM.docx]

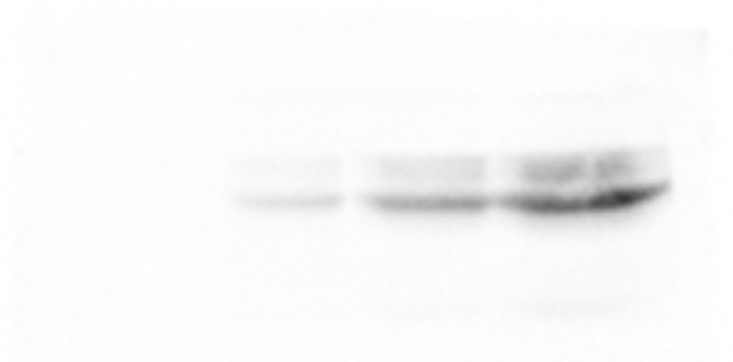


Figure 1b HTRA1


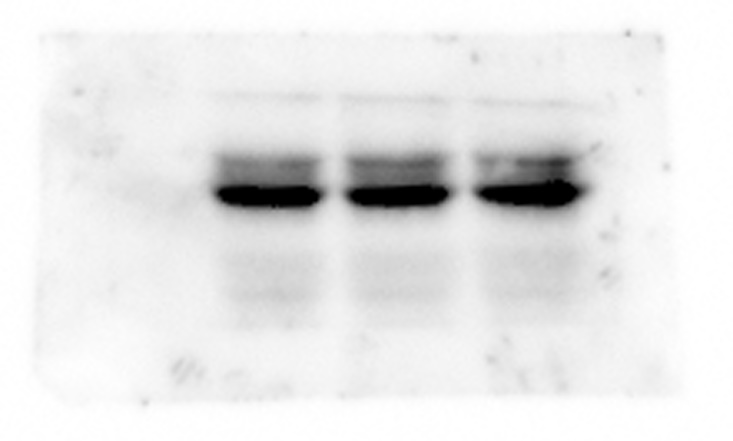


Figure 1b β-actin





Figure 2b SW1990 HTRA1





Figure 2b SW1990 β-actin





Figure 2b PANC-1 HTRA1





Figure 2b PANC-1 β-actin





Figure 3b SW1990 β-actin HTRA1





Figure 3b PANC1 β-actin HTRA1





Figure 5b Co-IP





Figure 5c sh β-actin





Figure 5c sh CDK1


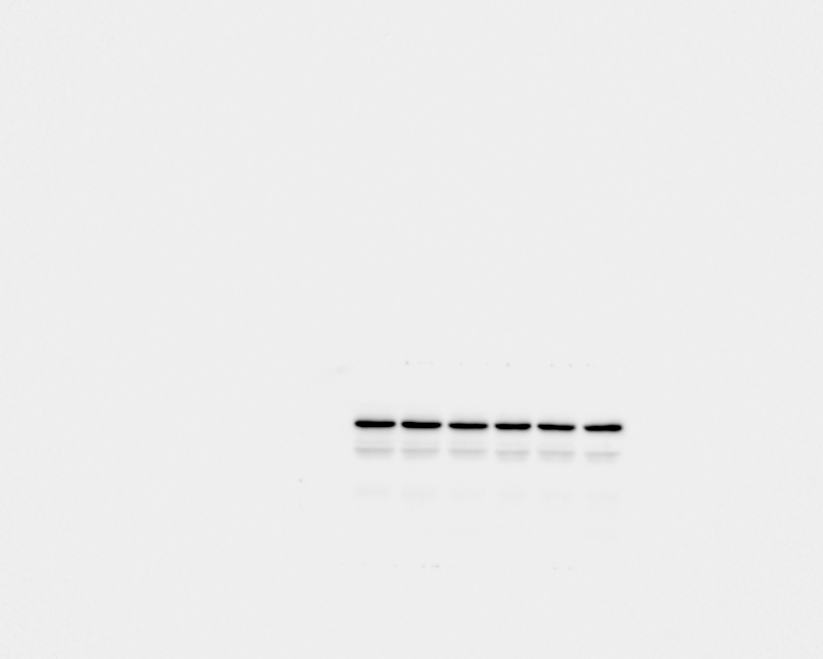


Figure 5c OE β-actin


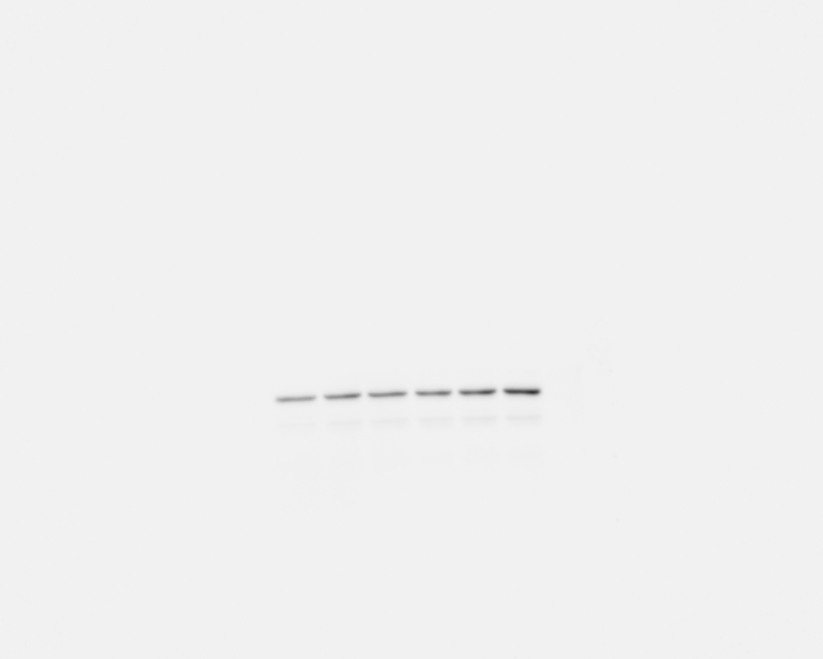


Figure 5c OE HTRA1


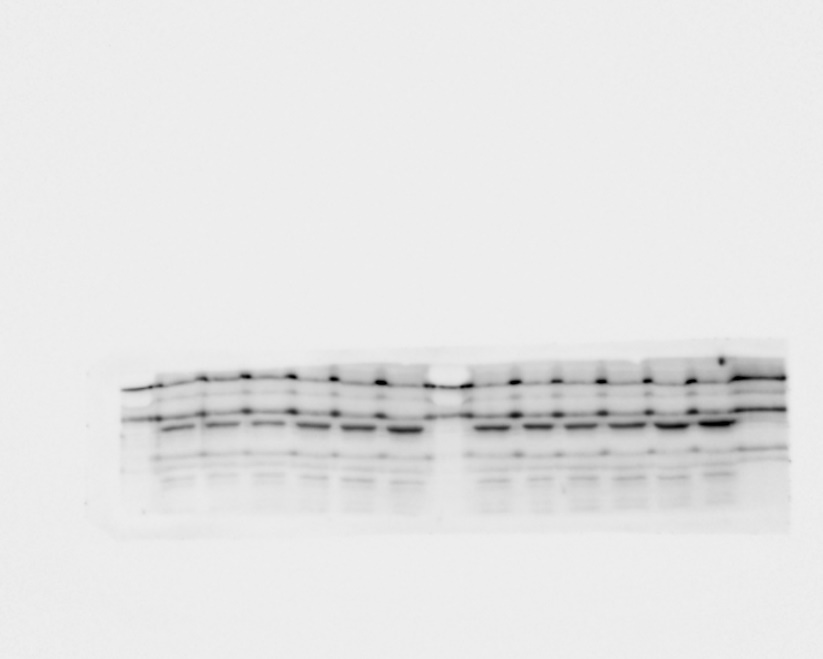


Figure 5c OE CDK1
