# Supplementary material for: Carfilzomib relieves pancreatitis-initiated pancreatic ductal adenocarcinoma by inhibiting high-temperature requirement protein A1
Source: Cell Death Discov. 2024 Jan 29;10:58. doi: 10.1038/s41420-024-01806-w (PMC10825157; doi:10.1038/s41420-024-01806-w)
Supplement: Supplementary file 2 — Supplementary [file 41420_2024_1806_MOESM2_ESM.docx]

**Supplementary Methods and Materials**

**Carfilzomib relieves Pancreatitis-initiated Pancreatic Ductal Adenocarcinoma by inhibiting High-Temperature Requirement Protein A1**

Fangyue Guo^1,2*^, Xufeng Tao^3*^, Yu Wu^1,2^, Deshi Dong^3^, Dong Shang^1,2,4#^, Hong Xiang^1#^

^1^ Laboratory of Integrative Medicine, First Affiliated Hospital of Dalian Medical University, Dalian, 116011, China.

^2^ Institute (College) of Integrative Medicine, Dalian Medical University, Dalian, 116044, China.

^3^ Department of Pharmacy, First Affiliated Hospital of Dalian Medical University, Dalian, 116011, China.

^4^ Department of General Surgery, First Affiliated Hospital of Dalian Medical University, Dalian, 116011, China.

^*^These authors contributed equally to this work.

^#^Corresponding author: Dong Shang, Laboratory of Integrative Medicine, First Affiliated Hospital of Dalian Medical University, Dalian, 116011, China; E-mail: [shangdong@dmu.edu.cn](mailto:shangdong@dmu.edu.cn). Hong Xiang, Laboratory of Integrative Medicine, First Affiliated Hospital of Dalian Medical University, Dalian, 116011, China; E-mail: [xianghong@dmu.edu.cn](mailto:xianghong@dmu.edu.cn).

**Supplementary Methods**

**Bioinformatics analysis**

Transcriptomic and clinical data for PDAC and normal pancreatic tissue were obtained from the Gene Expression Omnibus (GEO) and The Cancer Genome Atlas (TCGA) databases and The Genotype-Tissue Expression (GTEx). Following log2 transformation and batch correction, the TCGA cohort consisted of 150 PDAC and the GTEx consisted of 169 normal pancreatic samples, while the GSE15471 and GSE28735 cohorts each contained 39 pairs of pancreatic tumor and adjacent non-tumor tissue samples and 45 pairs of such samples, respectively.

**Immunohistochemistry**

A human tissue microarray of 80 paired PDAC patients (Cat No. PAC1601) was purchased from Shanghai Superbiotek Pharmaceutical Technology Co. Ltd. (Shanghai, China). Standard protocols were employed to perform HTRA1 staining, HTRA1 antibody (Abcam; Cambridge, UK，ab199529) was diluted of 1:1000，followed by quantitative analysis of the stained area using Image J software 1.51n. Then, using single-factor Cox regression analysis and Kaplan-Meier survival analysis, we investigated the prognostic significance of differential expression of HTRA1.

**Transfection experiments**

To identify the gene functions of *HTRA1* and *CDK1*, PANC-1 and SW1990 cells were seeded in 6-well plates for 48 h and then transfected for gene silencing. The transfected plasmids containing shRNA-*HTRA1* (shHTRA1) or shRNA-*CDK1* (sh*CDK1*) were used for gene silencing, and PANC-1 cells transfected with empty vector were used as a negative control (NC). Lentivirus was constructed for overexpress *HTRA1* gene, after 72 h of transfection, the cells was added to 8 μg/mL puromycin for 24 h to screen out the lentivirus-infected PANC-1 cell line. Pasmids and Lentivirus were purchased from GenePharma (Shanghai, China).

**Quantitative real-time PCR assay**

Total RNA was isolated from cultured PANC-1 and SW1990 cells using the TRNzol Universal total RNA extraction reagent (Accurate Biology, Hunan, China) according to the manufacturer’s instructions. RNA was reverse-transcribed by All-in-One First-Strand Synthesis MasterMix (with dsDNase) (Yugong Biolabs, Jiangsu, China) in a GeneExplorer PCR system (Bioer, Zhejiang, China), and the mRNA expressions were quantified using qPCR with Taq-HS SYBR® Green qPCR Premix (Universal) (Yugong Biolabs, Jiangsu, China) in an ABI 7500 Real-time PCR System (Applied Biosystems, Foster City, CA, USA). Amplification was performed at 95 ºC for 30 s, followed by 40 cycles of denaturing at 95 ºC for 5 s and annealing at 60 ºC for 34 s. The primer sequences were listed in Supplementary Table S1. All gene expression levels were normalized to β-actin, and the fold changes between the different groups were calculated using a standard curve for quantitative analysis.

**Flow cytometry**

Cell apoptosis was detected using an Annexin V-FITC/PI apoptosis detection kit (Meilun; Dalian, China). PANC-1 and SW1990 cells were cultured in 6-well plates adding different culture media for 48 h. Firstly, PANC-1 and SW1990 cells were digested with 0.25% trypsin and washed 2 times with PBS solution. Secondly, the collected cells were stained with 5 μL Annexin V-FITC plus 5 μL PI, and incubated at room temperature away from light for 15 mins. The stained cells were analyzed by flow cytometry (BD LSRFortessaTM Cell Analyzer).

The caspase-3 activity was determined using the Caspase-3 Fluorometric Assay Kit (ApexBio Technology; Houston, US). Briefly, PANC-1 and SW1990 cells were collected and after PBS washing, the cells were placed in a staining buffer with DEVD-AFC probe at 37°C for 1h. Following two additional washes with PBS, the cells were harvested, and caspase 3 activity was assessed using flow cytometry.

Cell cycle was measured using Cell Cycle Assay Kit (Meilun, Dalian, China) according to the manufacturer’s instructions. PANC-1 and SW1990 cells were harvested, washed with PBS, and fixed in 75% ethanol overnight at 4 °C. Prior to analysis, cells were washed again with PBS, resuspended and treated with DNase A 10 μL for 30 mins at 37 °C, and then incubated with 25 μL PI in the dark for 30 mins. Then the samples were analyzed by flow cytometry .

**Cell migration**

PANC-1 and SW1990 cells were cultured to 95% to 100% cell confluency in 6-well plates, and starved in a serum-free medium overnight. After the fresh medium was replaced, a single scratch in the cell monolayer of each well was created using a 200 μL pipet tip. Cell migration was imaged using a phase contrast microscope (Olympus Corp. Japan) at a magnification of 100× at 0 and 48 h post scratch. To create a reference point for repeated imaging of cell migration, a scratch was made on the bottom of the plate outside of the well, before plating the cells. The same region could be imaged over all the sampling times by this way.

**Cell invasion**

Cell invasion was assessed using a transwell with an 8 μm-pore of polycarbonate membranes coated by Matrigel matrix (Corning Inc., USA). Briefly, the cells (5 × 10^4^) were suspended in 100 μL of serum-free media and then added to the upper chamber. The lower chamber was supplemented with 800 μL containing 10% FBS. After 24 h, the invading cells were fixed with 10% methanol and stained with 0.1% crystal violet. The images were taken by microscope and the invading cells were counted. Crystal violet (Solarbio, Beijing, China) was diluted to 33% (v/v) with ddH2O. The bound crystal violet was eluted by adding 400 μL of 33% acetic acid into each insert and shaking for 10 mins. The eluent from the lower chamber was transferred to a 96-well clear microplate, and the absorbance at 590 nm was measured with a microplate reader (BioTek, US).

**Cell adhesion**

The 96-well plates were pretreated for 2 h with 0.5 mg/mL Matrigel matrix coated in medium containing 10% FBS. The concentration of cells in each group was adjusted to 5 × 10^5^/mL, and 100 μL/well was inoculated into 96-well plates. With 3 h incubation, the number of adherent cells was estimated by CCK-8 assay under a microplate reader (BioTek, US) at a wavelength of 450 nm.

**IP and LC-MS/MS analysis**

PANC-1 and SW1990 cells were harvested and lysed. Anti-DYKDDDDK Tag (Cell Signaling Technology, Massachusetts, USA) or IgG antibodies (Abcam; Cambridge, UK) were added to the lysis solution for antibody immobilization at 4 °C overnight. After incubation with Protein A/G Magnetic Beads (MedChemExpress; Shanghai, China) at 4 °C for 3 h, the protein complex was centrifuged and then washed 4 times with Pierce IP Lysis Buffer (Thermo; MA, USA) for SDS-PAGE analysis. Finally, the SDS-PAGE gel was subjected to silver staining to detect the difference in protein binding between the HTRA1 and IgG antibodies. In addition, after pull-down experiments, the two protein samples underwent reductive alkylation and enzymolysis. Moreover, to detect the polypeptide sequence of protein samples, LC-MS/MS analysis was implemented. The polypeptide sequence was identified using ProteinPilot software of the AB SCIEX Triple TOF™ 5600 plus MS system (MA, USA).

**Co-IP**

The lysates of PANC-1 cells were centrifuged at 12,000 rpm for 10 mins, and the supernatant was subsequently collected. DYKDDDDK antibody (10 μg) was then added to the remaining lysate for overnight incubation at 4 °C after a small amount of lysate was collected for other experiments. Protein A/G Magnetic Beads (MedChemExpress; Shanghai, China) were washed repeatedly by PBS with 0.5% triton, and incubated at room temperature for 2 h in the cell lysate. Supernatant and magnetic beads were then separated on a magnetic frame, and the beads were washed 3 times with 1 mL of Pierce IP Lysis Buffer (Thermo; MA, USA). Then, 100 μL of 2×SDS loading buffer was added, and samples were incubated at 95 °C for 5 min and subjected to western blot analysis.

**Western blot**

Total protein was extracted from PANC-1 and SW1990 cells using a protein extraction kit (KeyGEN BioTECH, Jiangsu, China), and the protein concentrations were determined using a BCA protein assay kit (KeyGEN BioTECH, Jiangsu, China). Protein samples were separated by SDS-PAGE (10-12%) and then transferred onto PVDF membranes (EMD Millipore). The membranes were blocked and incubated with primary antibodies (CDK1 ABclonal Technology Co.,Ltd, Wuhan,China A22347; HTRA1 Abcam; Cambridge, UK，ab199529. 1:1,000 dilution in 5% BSA) at 4 °C overnight. The membranes were then incubated with secondary antibodies at room temperature for 2 h. β‑actin (ABclonal Technology Co.,Ltd, Wuhan,China AC038. 1:50,000 dilution in 5% BSA) was used as the internal control. The protein expression in the membranes was visualized by enhanced ECL (Tanon Science and Technology) using Tanon‑5200 Multi Gel Imaging System (Tanon Science and Technology). Quantitative analysis was performed using Gel‑Pro analyzer 4.0 software (Media Cybernetics).

**Pancreatic section staining**

Tissues were fixed in 4% paraformaldehyde overnight and embedded in paraffin . Next, pancreatic tissues were serially sectioned at the thickness of 3 μm and stained with HTRA1 staining, hematoxylin and eosin, Alcian blue, and Sirius red. Image J software 1.51n was utilized to quantify total pancreatic area, Alcian blue-positive low-stage PanIN area and Sirius red affected area.

**Virtual screening**

The dock module in MOE(14) was used for structure-based virtual screening (SBVS). About 2800 approved drug molecules at Drugbank were selected as virtual screening (VS) library. All compounds were prepared with the Wash module in MOE. The structure of HTRA1 was download from RCSB PDB Data Bank with PDB ID of 3NZI2. The molecule binding site around HTRA1 of residue Leu307, Val347, Leu309, Leu345, Lys346, Thr344, Ser328, Asp250, His220, Tyr325, Asn327, Ile323, Asn324 and Asn343 was selected as binding pocket. After that all compounds were ranked by the flexible docking with the “induced fit” protocol. Prior to docking, the force field of AMBER10: EHT and the implicit solvation model of Reaction Field (R-field) were selected. The protonation state of the protein and the orientation of the hydrogens were optimized by QuickPrep module at the PH of 7 and temperature of 300 K. For flexible docking, the docked poses were ranked by London dG scoring first, then a force field refinement was carried out on the Top 10 poses followed by a rescoring of GBVI/WSA dG and the best ranked pose was retained. After docking, the compounds were clustered structurally through the Fingerprint Cluster module in MOE. The best ranked 20 molecules were finally identified as potential hits.

**Pharmacodynamic validation**

To evaluate the therapeutic effect of candidate small molecule inhibitors, we first determined their toxicity with the CCK-8 assay, followed by apoptosis, cell cycle, migration, invasion and adhesion of PANC-1 and SW1990 cells with the corresponding kits. In addition, we investigated pancreatic pathology in KC mice by performing HE staining, Alcian blue staining and Sirius red staining.

Supplementary Table S1 Unique proteins of pull-down in HTRA1 IP group.

| Accession | Names | Length | Mass | Unused | Coverage (%) |
| --- | --- | --- | --- | --- | --- |
| sp\|Q5VTE0\|EF1A3_HUMAN | Putative elongation factor 1-alpha-like 3 | 462 | 50184.7 | 11.32 | 21.43000066 |
| sp\|P10768\|ESTD_HUMAN | S-formylglutathione hydrolase | 282 | 31462.5 | 3.15 | 9.573999792 |
| sp\|Q9H936\|GHC1_HUMAN | Mitochondrial glutamate carrier 1 | 323 | 34469.8 | 5.1 | 11.15000024 |
| sp\|P84098\|RL19_HUMAN | 60S ribosomal protein L19 | 196 | 23465.8 | 5.2 | 22.45000005 |
| sp\|P04179\|SODM_HUMAN | Superoxide dismutase [Mn], mitochondrial | 222 | 24750 | 3.19 | 10.36000028 |
| sp\|P51571\|SSRD_HUMAN | Translocon-associated protein subunit delta | 173 | 18998.4 | 4 | 17.33999997 |
| sp\|Q04917\|1433F_HUMAN | 14-3-3 protein eta | 246 | 28218.4 | 5.14 | 19.11000013 |
| sp\|P28074\|PSB5_HUMAN | Proteasome subunit beta type-5 | 263 | 28480 | 4.1 | 11.41000018 |
| sp\|P09211\|GSTP1_HUMAN | Glutathione S-transferase P | 210 | 23355.6 | 15.35 | 49.05000031 |
| sp\|Q9BQE3\|TBA1C_HUMAN | Tubulin alpha-1C chain | 449 | 49894.9 | 12.04 | 19.38000023 |
| sp\|P09429\|HMGB1_HUMAN | High mobility group protein B1 | 215 | 24893.6 | 4.21 | 11.63000017 |
| sp\|Q9Y3A5\|SBDS_HUMAN | Ribosome maturation protein SBDS | 250 | 28763.3 | 4.02 | 10.40000021 |
| sp\|P07737\|PROF1_HUMAN | Profilin-1 | 140 | 15054.1 | 12 | 51.42999887 |
| sp\|P62701\|RS4X_HUMAN | 40S ribosomal protein S4, X isoform | 263 | 29597.5 | 15.57 | 26.6200006 |
| sp\|P0DMV9\|HS71B_HUMAN | Heat shock 70 kDa protein 1B | 641 | 70051.7 | 4.25 | 4.523999989 |
| sp\|P59998\|ARPC4_HUMAN | Actin-related protein 2/3 complex subunit 4 | 168 | 19666.8 | 3.09 | 11.30999997 |
| sp\|P38117\|ETFB_HUMAN | Electron transfer flavoprotein subunit beta | 255 | 27843.4 | 2.6 | 8.626999706 |
| sp\|P20290\|BTF3_HUMAN | Transcription factor BTF3 | 206 | 22167.8 | 1.44 | 3.398000076 |
| sp\|P46782\|RS5_HUMAN | 40S ribosomal protein S5 | 204 | 22876.2 | 14.75 | 37.25000024 |
| sp\|P23919\|KTHY_HUMAN | Thymidylate kinase | 212 | 23819.1 | 2 | 5.18900007 |
| sp\|P22626\|ROA2_HUMAN | Heterogeneous nuclear ribonucleoproteins A2/B1 | 353 | 37429.7 | 8.17 | 17.56000072 |
| sp\|P07437\|TBB5_HUMAN | Tubulin beta chain | 444 | 49670.5 | 5.77 | 8.558999747 |
| sp\|P16070\|CD44_HUMAN | CD44 antigen | 742 | 81537 | 2 | 1.75199993 |
| sp\|Q96C19\|EFHD2_HUMAN | EF-hand domain-containing protein D2 | 240 | 26697 | 2 | 7.083000243 |
| sp\|P27348\|1433T_HUMAN | 14-3-3 protein theta | 245 | 27763.9 | 23.38 | 47.35000134 |
| sp\|Q9HB71\|CYBP_HUMAN | Calcyclin-binding protein | 228 | 26209.8 | 5.89 | 17.54000038 |
| sp\|P13995\|MTDC_HUMAN | Bifunctional methylenetetrahydrofolate dehydrogenase/cyclohydrolase, mitochondrial | 350 | 37894.8 | 2.18 | 6.571000069 |
| sp\|P18669\|PGAM1_HUMAN | Phosphoglycerate mutase 1 | 254 | 28803.7 | 17.65 | 40.54999948 |
| sp\|P62269\|RS18_HUMAN | 40S ribosomal protein S18 | 152 | 17718.6 | 6.6 | 15.79000056 |
| sp\|O15511\|ARPC5_HUMAN | Actin-related protein 2/3 complex subunit 5 | 151 | 16320.3 | 1.52 | 7.947000116 |
| sp\|P21964\|COMT_HUMAN | Catechol O-methyltransferase | 271 | 30036.8 | 2 | 5.90399988 |
| sp\|P61313\|RL15_HUMAN | 60S ribosomal protein L15 | 204 | 24145.9 | 3.68 | 13.24000061 |
| sp\|Q13595\|TRA2A_HUMAN | Transformer-2 protein homolog alpha | 282 | 32688.3 | 2 | 4.964999855 |
| sp\|Q99880\|H2B1L_HUMAN | Histone H2B type 1-L | 126 | 13952.1 | 6.92 | 23.81000072 |
| sp\|P08648\|ITA5_HUMAN | Integrin alpha-5 | 1049 | 114535.5 | 1.72 | 1.049000025 |
| sp\|P31946\|1433B_HUMAN | 14-3-3 protein beta/alpha | 246 | 28082.2 | 9.81 | 39.84000087 |
| sp\|P37802\|TAGL2_HUMAN | Transgelin-2 | 199 | 22391.4 | 12.59 | 40.70000052 |
| sp\|O75489\|NDUS3_HUMAN | NADH dehydrogenase [ubiquinone] iron-sulfur protein 3, mitochondrial | 264 | 30241.2 | 3.54 | 9.848000109 |
| sp\|Q15185\|TEBP_HUMAN | Prostaglandin E synthase 3 | 160 | 18697.2 | 4.38 | 14.37000036 |
| sp\|P21291\|CSRP1_HUMAN | Cysteine and glycine-rich protein 1 | 193 | 20567.3 | 2 | 7.772000134 |
| sp\|Q13155\|AIMP2_HUMAN | Aminoacyl tRNA synthase complex-interacting multifunctional protein 2 | 320 | 35348.5 | 1.96 | 4.061999917 |
| sp\|Q96PK6\|RBM14_HUMAN | RNA-binding protein 14 | 669 | 69490.9 | 1.48 | 2.39199996 |
| sp\|Q96AG4\|LRC59_HUMAN | Leucine-rich repeat-containing protein 59 | 307 | 34930.1 | 6.64 | 12.3800002 |
| sp\|Q02878\|RL6_HUMAN | 60S ribosomal protein L6 | 288 | 32727.7 | 9.23 | 15.27999938 |
| sp\|Q14165\|MLEC_HUMAN | Malectin | 292 | 32233.6 | 3.74 | 8.562000096 |
| sp\|P06493\|CDK1_HUMAN | Cyclin-dependent kinase 1 | 297 | 34095.1 | 7.58 | 14.13999945 |
| sp\|O00233\|PSMD9_HUMAN | 26S proteasome non-ATPase regulatory subunit 9 | 223 | 24681.7 | 1.77 | 5.381000042 |
| sp\|P13987\|CD59_HUMAN | CD59 glycoprotein | 128 | 14177.2 | 3.15 | 20.30999959 |
| sp\|Q06323\|PSME1_HUMAN | Proteasome activator complex subunit 1 | 249 | 28722.9 | 1.76 | 5.621999875 |
| sp\|P61019\|RAB2A_HUMAN | Ras-related protein Rab-2A | 212 | 23545.4 | 3.01 | 11.7899999 |
| sp\|P54819\|KAD2_HUMAN | Adenylate kinase 2, mitochondrial | 239 | 26477.4 | 6.52 | 16.74000025 |
| sp\|P40925\|MDHC_HUMAN | Malate dehydrogenase, cytoplasmic | 334 | 36425.8 | 9.57 | 22.15999961 |
| sp\|P62888\|RL30_HUMAN | 60S ribosomal protein L30 | 115 | 12783.9 | 6.69 | 26.08999908 |
| sp\|P37837\|TALDO_HUMAN | Transaldolase | 337 | 37539.7 | 8.44 | 15.72999954 |
| sp\|P49720\|PSB3_HUMAN | Proteasome subunit beta type-3 | 205 | 22948.7 | 2 | 7.805000246 |
| sp\|P61981\|1433G_HUMAN | 14-3-3 protein gamma | 247 | 28302.3 | 8.59 | 30.77000082 |
| sp\|P39687\|AN32A_HUMAN | Acidic leucine-rich nuclear phosphoprotein 32 family member A | 249 | 28585.1 | 4.36 | 8.8349998 |
| sp\|Q9UBQ7\|GRHPR_HUMAN | Glyoxylate reductase/hydroxypyruvate reductase | 328 | 35667.9 | 4.9 | 12.5 |
| sp\|P08670\|VIME_HUMAN | Vimentin | 466 | 53651.2 | 2.76 | 7.511000335 |
| sp\|P61224\|RAP1B_HUMAN | Ras-related protein Rap-1b | 184 | 20824.7 | 5.69 | 19.57000047 |
| sp\|Q9H9B4\|SFXN1_HUMAN | Sideroflexin-1 | 322 | 35619.1 | 2.92 | 8.384999633 |
| sp\|O14818\|PSA7_HUMAN | Proteasome subunit alpha type-7 | 248 | 27886.6 | 8.3 | 20.97000033 |
| sp\|P39019\|RS19_HUMAN | 40S ribosomal protein S19 | 145 | 16060.4 | 5.96 | 22.06999958 |
| sp\|P29218\|IMPA1_HUMAN | Inositol monophosphatase 1 | 277 | 30188.6 | 4.77 | 9.747000039 |
| sp\|P40926\|MDHM_HUMAN | Malate dehydrogenase, mitochondrial | 338 | 35502.9 | 43.41 | 63.02000284 |
| sp\|Q15785\|TOM34_HUMAN | Mitochondrial import receptor subunit TOM34 | 309 | 34559 | 1.8 | 6.471999735 |
| sp\|P18621\|RL17_HUMAN | 60S ribosomal protein L17 | 184 | 21396.9 | 2.91 | 10.32999977 |
| sp\|P62258\|1433E_HUMAN | 14-3-3 protein epsilon | 255 | 29173.6 | 8.9 | 36.0799998 |
| sp\|O95994\|AGR2_HUMAN | Anterior gradient protein 2 homolog | 175 | 19979 | 1.81 | 6.285999715 |
| sp\|P05198\|IF2A_HUMAN | Eukaryotic translation initiation factor 2 subunit 1 | 315 | 36111.8 | 2 | 3.810000047 |
| sp\|P81605\|DCD_HUMAN | Dermcidin | 110 | 11283.7 | 1.59 | 22.73000032 |
| sp\|P09525\|ANXA4_HUMAN | Annexin A4 | 319 | 35882.4 | 13.16 | 28.83999944 |
| sp\|Q9UNX3\|RL26L_HUMAN | 60S ribosomal protein L26-like 1 | 145 | 17256.2 | 4.71 | 11.0299997 |
| sp\|P04632\|CPNS1_HUMAN | Calpain small subunit 1 | 268 | 28315.6 | 4.06 | 9.328000247 |
| sp\|P24534\|EF1B_HUMAN | Elongation factor 1-beta | 225 | 24763.5 | 2.13 | 9.777999669 |
| sp\|P68402\|PA1B2_HUMAN | Platelet-activating factor acetylhydrolase IB subunit beta | 229 | 25569.1 | 2.01 | 3.929999843 |
| sp\|P21796\|VDAC1_HUMAN | Voltage-dependent anion-selective channel protein 1 | 283 | 30772.4 | 5.14 | 15.18999934 |
| sp\|P42766\|RL35_HUMAN | 60S ribosomal protein L35 | 123 | 14551.4 | 3.41 | 18.70000064 |
| sp\|P20618\|PSB1_HUMAN | Proteasome subunit beta type-1 | 241 | 26489.1 | 3.69 | 9.959000349 |
| sp\|P60900\|PSA6_HUMAN | Proteasome subunit alpha type-6 | 246 | 27399.2 | 6.36 | 13.00999969 |
| sp\|P46781\|RS9_HUMAN | 40S ribosomal protein S9 | 194 | 22591.2 | 9.74 | 14.94999975 |
| sp\|Q96C36\|P5CR2_HUMAN | Pyrroline-5-carboxylate reductase 2 | 320 | 33636.8 | 5.42 | 12.5 |
| sp\|Q9UL46\|PSME2_HUMAN | Proteasome activator complex subunit 2 | 239 | 27401.4 | 2.66 | 10.45999974 |
| sp\|P32119\|PRDX2_HUMAN | Peroxiredoxin-2 | 198 | 21891.7 | 1.37 | 17.67999977 |
| sp\|P62910\|RL32_HUMAN | 60S ribosomal protein L32 | 135 | 15859.7 | 3.67 | 14.81000036 |
| sp\|P60660\|MYL6_HUMAN | Myosin light polypeptide 6 | 151 | 16930 | 2.03 | 8.608999848 |
| sp\|Q53GQ0\|DHB12_HUMAN | Very-long-chain 3-oxoacyl-CoA reductase | 312 | 34323.9 | 2.19 | 4.808000103 |
| sp\|P08134\|RHOC_HUMAN | Rho-related GTP-binding protein RhoC | 193 | 22006.2 | 3.55 | 11.91999987 |
| sp\|P62277\|RS13_HUMAN | 40S ribosomal protein S13 | 151 | 17222.1 | 3.26 | 8.608999848 |
| sp\|P62249\|RS16_HUMAN | 40S ribosomal protein S16 | 146 | 16445.2 | 11.52 | 25.33999979 |
| sp\|Q15181\|IPYR_HUMAN | Inorganic pyrophosphatase | 289 | 32659.8 | 2.56 | 3.113999963 |
| sp\|P14174\|MIF_HUMAN | Macrophage migration inhibitory factor | 115 | 12476.2 | 4 | 17.38999933 |
| sp\|P62316\|SMD2_HUMAN | Small nuclear ribonucleoprotein Sm D2 | 118 | 13526.8 | 2.05 | 8.474999666 |
| sp\|P62937\|PPIA_HUMAN | Peptidyl-prolyl cis-trans isomerase A | 165 | 18012.4 | 19.11 | 61.82000041 |
| sp\|P62873\|GBB1_HUMAN | Guanine nucleotide-binding protein G(I)/G(S)/G(T) subunit beta-1 | 340 | 37376.6 | 2 | 5.882000178 |
| sp\|P61758\|PFD3_HUMAN | Prefoldin subunit 3 | 197 | 22657.8 | 2.04 | 5.076000094 |
| sp\|P40429\|RL13A_HUMAN | 60S ribosomal protein L13a | 203 | 23577.1 | 2.9 | 11.33000031 |
| sp\|P08758\|ANXA5_HUMAN | Annexin A5 | 320 | 35936.4 | 26.66 | 45.30999959 |
| sp\|P27105\|STOM_HUMAN | Erythrocyte band 7 integral membrane protein | 288 | 31730.4 | 3.66 | 12.15000004 |
| sp\|P62241\|RS8_HUMAN | 40S ribosomal protein S8 | 208 | 24205 | 11.08 | 32.69000053 |
| sp\|P84095\|RHOG_HUMAN | Rho-related GTP-binding protein RhoG | 191 | 21308.3 | 5.82 | 36.64999902 |
| sp\|P84103\|SRSF3_HUMAN | Serine/arginine-rich splicing factor 3 | 164 | 19329.4 | 4.12 | 23.17000031 |
| sp\|P25786\|PSA1_HUMAN | Proteasome subunit alpha type-1 | 263 | 29555.3 | 5.9 | 14.45000023 |
| sp\|P28066\|PSA5_HUMAN | Proteasome subunit alpha type-5 | 241 | 26410.8 | 3.68 | 14.11000043 |
| sp\|P04792\|HSPB1_HUMAN | Heat shock protein beta-1 | 205 | 22782.3 | 9.54 | 29.76000011 |
| sp\|P13693\|TCTP_HUMAN | Translationally-controlled tumor protein | 172 | 19595.2 | 2 | 7.558000088 |
| sp\|Q96QV6\|H2A1A_HUMAN | Histone H2A type 1-A | 131 | 14233.4 | 4 | 21.36999965 |
| sp\|P47914\|RL29_HUMAN | 60S ribosomal protein L29 | 159 | 17751.9 | 2.88 | 9.433999658 |
| sp\|P07339\|CATD_HUMAN | Cathepsin D | 412 | 44551.8 | 14.22 | 22.08999991 |
| sp\|P26373\|RL13_HUMAN | 60S ribosomal protein L13 | 211 | 24261.3 | 11.16 | 22.75000066 |
| sp\|Q13185\|CBX3_HUMAN | Chromobox protein homolog 3 | 183 | 20811.2 | 1.92 | 7.649999857 |
| sp\|Q8NCW5\|NNRE_HUMAN | NAD(P)H-hydrate epimerase | 288 | 31674.3 | 2 | 6.25 |
| sp\|Q04837\|SSBP_HUMAN | Single-stranded DNA-binding protein, mitochondrial | 148 | 17259.6 | 6.58 | 37.83999979 |
| sp\|P30048\|PRDX3_HUMAN | Thioredoxin-dependent peroxide reductase, mitochondrial | 256 | 27692.4 | 5.46 | 10.15999988 |
| sp\|P60866\|RS20_HUMAN | 40S ribosomal protein S20 | 119 | 13372.6 | 7.9 | 31.09000027 |
| sp\|P23284\|PPIB_HUMAN | Peptidyl-prolyl cis-trans isomerase B | 216 | 23742.4 | 11.24 | 27.77999938 |
| sp\|P60174\|TPIS_HUMAN | Triosephosphate isomerase | 286 | 30790.8 | 23.63 | 55.58999777 |
| sp\|Q9NR31\|SAR1A_HUMAN | GTP-binding protein SAR1a | 198 | 22366.6 | 2.42 | 5.556000024 |
| sp\|Q15366\|PCBP2_HUMAN | Poly(rC)-binding protein 2 | 365 | 38579.7 | 3.38 | 10.96000001 |
| sp\|P07741\|APT_HUMAN | Adenine phosphoribosyltransferase | 180 | 19607.5 | 10 | 34.43999887 |
| sp\|P47756\|CAPZB_HUMAN | F-actin-capping protein subunit beta | 277 | 31350.2 | 2.11 | 8.664000034 |
| sp\|Q9BVC6\|TM109_HUMAN | Transmembrane protein 109 | 243 | 26209.6 | 2.29 | 4.938000068 |
| sp\|O00487\|PSDE_HUMAN | 26S proteasome non-ATPase regulatory subunit 14 | 310 | 34576.9 | 2 | 4.193999991 |
| sp\|P63027\|VAMP2_HUMAN | Vesicle-associated membrane protein 2 | 116 | 12662.6 | 4.16 | 20.69000006 |
| sp\|P12004\|PCNA_HUMAN | Proliferating cell nuclear antigen | 261 | 28768.5 | 17.38 | 42.53000021 |
| sp\|P19623\|SPEE_HUMAN | Spermidine synthase | 302 | 33824.5 | 3.36 | 5.959999934 |
| sp\|Q99627\|CSN8_HUMAN | COP9 signalosome complex subunit 8 | 209 | 23225.4 | 1.8 | 6.699000299 |
| sp\|P62424\|RL7A_HUMAN | 60S ribosomal protein L7a | 266 | 29995.4 | 21.09 | 36.46999896 |
| sp\|Q99623\|PHB2_HUMAN | Prohibitin-2 | 299 | 33296.1 | 4.23 | 7.357999682 |
| sp\|Q16629\|SRSF7_HUMAN | Serine/arginine-rich splicing factor 7 | 238 | 27366.2 | 2.97 | 10.92000008 |
| sp\|P62854\|RS26_HUMAN | 40S ribosomal protein S26 | 115 | 13015.3 | 2.21 | 13.0400002 |
| sp\|P48047\|ATPO_HUMAN | ATP synthase subunit O, mitochondrial | 213 | 23277.1 | 7.35 | 24.87999946 |
| sp\|Q9NQ39\|RS10L_HUMAN | Putative 40S ribosomal protein S10-like | 176 | 20120.2 | 2 | 7.954999804 |
| sp\|P09601\|HMOX1_HUMAN | Heme oxygenase 1 | 288 | 32818.3 | 2 | 8.332999796 |
| sp\|P08708\|RS17_HUMAN | 40S ribosomal protein S17 | 135 | 15550 | 4.01 | 8.889000118 |
| sp\|P62851\|RS25_HUMAN | 40S ribosomal protein S25 | 125 | 13742 | 1.92 | 7.999999821 |
| sp\|P25787\|PSA2_HUMAN | Proteasome subunit alpha type-2 | 234 | 25898.4 | 6 | 20.93999982 |
| sp\|P62753\|RS6_HUMAN | 40S ribosomal protein S6 | 249 | 28680.4 | 12.54 | 29.71999943 |
| sp\|Q15717\|ELAV1_HUMAN | ELAV-like protein 1 | 326 | 36091.6 | 3.49 | 7.361999899 |
| sp\|P60709\|ACTB_HUMAN | Actin, cytoplasmic 1 | 375 | 41736.4 | 15.48 | 21.07000053 |
| sp\|Q16698\|DECR_HUMAN | 2,4-dienoyl-CoA reductase, mitochondrial | 335 | 36067.4 | 5.55 | 13.42999935 |
| sp\|P23528\|COF1_HUMAN | Cofilin-1 | 166 | 18502.3 | 17.72 | 50 |
| sp\|Q71DI3\|H32_HUMAN | Histone H3.2 | 136 | 15387.9 | 4.53 | 11.76000014 |
| sp\|P54920\|SNAA_HUMAN | Alpha-soluble NSF attachment protein | 295 | 33232.3 | 2.77 | 7.796999812 |
| sp\|Q9Y3B3\|TMED7_HUMAN | Transmembrane emp24 domain-containing protein 7 | 224 | 25171.4 | 2.73 | 10.71000025 |
| sp\|P62826\|RAN_HUMAN | GTP-binding nuclear protein Ran | 216 | 24423 | 20.78 | 36.57000065 |
| sp\|P46777\|RL5_HUMAN | 60S ribosomal protein L5 | 297 | 34362.4 | 8.31 | 19.52999979 |
| sp\|P61289\|PSME3_HUMAN | Proteasome activator complex subunit 3 | 254 | 29505.8 | 3.87 | 10.23999974 |
| sp\|P61026\|RAB10_HUMAN | Ras-related protein Rab-10 | 200 | 22540.7 | 4.31 | 22.4999994 |
| sp\|P60763\|RAC3_HUMAN | Ras-related C3 botulinum toxin substrate 3 | 192 | 21378.7 | 2.03 | 10.93999967 |
| sp\|O95816\|BAG2_HUMAN | BAG family molecular chaperone regulator 2 | 211 | 23771.7 | 2.2 | 5.21299988 |
| sp\|P62263\|RS14_HUMAN | 40S ribosomal protein S14 | 151 | 16272.6 | 7.86 | 39.73999918 |
| sp\|P28072\|PSB6_HUMAN | Proteasome subunit beta type-6 | 239 | 25357.5 | 3.41 | 4.602999985 |
| sp\|P02786\|TFR1_HUMAN | Transferrin receptor protein 1 | 760 | 84870.7 | 2 | 2.105000056 |
| sp\|P46779\|RL28_HUMAN | 60S ribosomal protein L28 | 137 | 15747.4 | 8.89 | 34.31000113 |
| sp\|Q3MHD2\|LSM12_HUMAN | Protein LSM12 homolog | 195 | 21700.6 | 1.85 | 6.667000055 |
| sp\|Q15560\|TCEA2_HUMAN | Transcription elongation factor A protein 2 | 299 | 33600.4 | 2 | 4.348000139 |
| sp\|P22087\|FBRL_HUMAN | rRNA 2'-O-methyltransferase fibrillarin | 321 | 33784.1 | 8.9 | 20.87000012 |
| sp\|P78417\|GSTO1_HUMAN | Glutathione S-transferase omega-1 | 241 | 27565.6 | 4.11 | 9.959000349 |
| sp\|Q3ZCQ8\|TIM50_HUMAN | Mitochondrial import inner membrane translocase subunit TIM50 | 353 | 39645.8 | 19.59 | 22.10000008 |
| sp\|P30040\|ERP29_HUMAN | Endoplasmic reticulum resident protein 29 | 261 | 28993.2 | 5.24 | 14.18000013 |
| sp\|Q03135\|CAV1_HUMAN | Caveolin-1 | 178 | 20471.4 | 2.29 | 4.493999854 |
| sp\|Q9NUQ9\|FA49B_HUMAN | Protein FAM49B | 324 | 36747.7 | 2 | 5.863999948 |
| sp\|P25788\|PSA3_HUMAN | Proteasome subunit alpha type-3 | 255 | 28433 | 7.68 | 21.17999941 |
| sp\|P50402\|EMD_HUMAN | Emerin | 254 | 28993.5 | 4 | 10.62999964 |
| sp\|Q9BRL6\|SRSF8_HUMAN | Serine/arginine-rich splicing factor 8 | 282 | 32287.1 | 1.78 | 2.837000042 |
| sp\|P27635\|RL10_HUMAN | 60S ribosomal protein L10 | 214 | 24603.7 | 3.42 | 8.410999924 |
| sp\|P62913\|RL11_HUMAN | 60S ribosomal protein L11 | 178 | 20252.2 | 4.24 | 12.91999966 |
| sp\|P23396\|RS3_HUMAN | 40S ribosomal protein S3 | 243 | 26688.1 | 27.09 | 60.07999778 |
| sp\|P06733\|ENOA_HUMAN | Alpha-enolase | 434 | 47168.6 | 4.28 | 6.221000105 |
| sp\|P30041\|PRDX6_HUMAN | Peroxiredoxin-6 | 224 | 25034.7 | 5.22 | 19.64000016 |
| sp\|P63104\|1433Z_HUMAN | 14-3-3 protein zeta/delta | 245 | 27744.8 | 27.44 | 58.78000259 |
| sp\|Q9H0U4\|RAB1B_HUMAN | Ras-related protein Rab-1B | 201 | 22171 | 16.92 | 40.29999971 |
| sp\|P51148\|RAB5C_HUMAN | Ras-related protein Rab-5C | 216 | 23482.4 | 8.5 | 29.17000055 |
| sp\|P15880\|RS2_HUMAN | 40S ribosomal protein S2 | 293 | 31324.2 | 9.67 | 14.32999969 |
| sp\|Q9BS40\|LXN_HUMAN | Latexin | 222 | 25750.1 | 4 | 12.16000021 |
| sp\|P55795\|HNRH2_HUMAN | Heterogeneous nuclear ribonucleoprotein H2 | 449 | 49263.3 | 2.01 | 3.785999864 |
| sp\|P13073\|COX41_HUMAN | Cytochrome c oxidase subunit 4 isoform 1, mitochondrial | 169 | 19576.6 | 1.34 | 6.509000063 |
| sp\|P22061\|PIMT_HUMAN | Protein-L-isoaspartate(D-aspartate) O-methyltransferase | 227 | 24636.2 | 2.68 | 7.489000261 |
| sp\|Q15691\|MARE1_HUMAN | Microtubule-associated protein RP/EB family member 1 | 268 | 29998.9 | 7.32 | 26.87000036 |
| sp\|Q5JXB2\|UE2NL_HUMAN | Putative ubiquitin-conjugating enzyme E2 N-like | 153 | 17376.8 | 2.08 | 7.190000266 |
| sp\|P45880\|VDAC2_HUMAN | Voltage-dependent anion-selective channel protein 2 | 294 | 31566.3 | 6.07 | 14.2900005 |
| sp\|P53597\|SUCA_HUMAN | Succinate--CoA ligase [ADP/GDP-forming] subunit alpha, mitochondrial | 346 | 36249.5 | 1.74 | 4.623999819 |
| sp\|P62829\|RL23_HUMAN | 60S ribosomal protein L23 | 140 | 14865.3 | 6.18 | 32.85999894 |
| sp\|P35268\|RL22_HUMAN | 60S ribosomal protein L22 | 128 | 14786.9 | 7.77 | 41.40999913 |
| sp\|P38646\|GRP75_HUMAN | Stress-70 protein, mitochondrial | 679 | 73680 | 2.21 | 3.829000145 |
| sp\|O43291\|SPIT2_HUMAN | Kunitz-type protease inhibitor 2 | 252 | 28227.9 | 2 | 4.761999846 |
| sp\|Q9NQR4\|NIT2_HUMAN | Omega-amidase NIT2 | 276 | 30607.6 | 6 | 15.57999998 |
| sp\|P47755\|CAZA2_HUMAN | F-actin-capping protein subunit alpha-2 | 286 | 32948.9 | 3.31 | 13.98999989 |
| sp\|P04083\|ANXA1_HUMAN | Annexin A1 | 346 | 38713.9 | 24.06 | 34.97000039 |
| sp\|P56537\|IF6_HUMAN | Eukaryotic translation initiation factor 6 | 245 | 26598.8 | 2.39 | 5.714000016 |
| sp\|P07355\|ANXA2_HUMAN | Annexin A2 | 339 | 38603.6 | 57.76 | 67.84999967 |
| sp\|P21266\|GSTM3_HUMAN | Glutathione S-transferase Mu 3 | 225 | 26559.3 | 4.54 | 9.333000332 |
| sp\|Q15102\|PA1B3_HUMAN | Platelet-activating factor acetylhydrolase IB subunit gamma | 231 | 25734.1 | 4.59 | 11.25999987 |
| sp\|Q5JWF2\|GNAS1_HUMAN | Guanine nucleotide-binding protein G(s) subunit alpha isoforms XLas | 1037 | 111023.3 | 1.32 | 1.061000023 |
| sp\|Q07955\|SRSF1_HUMAN | Serine/arginine-rich splicing factor 1 | 248 | 27744.3 | 5.52 | 13.3100003 |
| sp\|P17931\|LEG3_HUMAN | Galectin-3 | 250 | 26152.2 | 1.6 | 4.399999976 |
| sp\|P62917\|RL8_HUMAN | 60S ribosomal protein L8 | 257 | 28024.5 | 8.23 | 18.68000031 |
| sp\|O43809\|CPSF5_HUMAN | Cleavage and polyadenylation specificity factor subunit 5 | 227 | 26227.1 | 1.72 | 3.965000063 |
| sp\|P62993\|GRB2_HUMAN | Growth factor receptor-bound protein 2 | 217 | 25206.2 | 1.7 | 6.452000141 |
| sp\|P07195\|LDHB_HUMAN | L-lactate dehydrogenase B chain | 334 | 36638.2 | 13.39 | 32.62999952 |
| sp\|P16402\|H13_HUMAN | Histone H1.3 | 221 | 22349.7 | 6.97 | 17.19000041 |
| sp\|P26006\|ITA3_HUMAN | Integrin alpha-3 | 1051 | 116611.3 | 3.51 | 3.139999881 |
| sp\|O15144\|ARPC2_HUMAN | Actin-related protein 2/3 complex subunit 2 | 300 | 34332.7 | 2.53 | 12.6699999 |
| sp\|P18124\|RL7_HUMAN | 60S ribosomal protein L7 | 248 | 29225.6 | 16.21 | 30.23999929 |
| sp\|P60953\|CDC42_HUMAN | Cell division control protein 42 homolog | 191 | 21258.4 | 9.71 | 36.64999902 |
| sp\|P35232\|PHB_HUMAN | Prohibitin | 272 | 29803.8 | 5.33 | 15.07000029 |
| sp\|P63244\|RACK1_HUMAN | Receptor of activated protein C kinase 1 | 317 | 35076.5 | 21.24 | 46.6899991 |
| sp\|Q3ZAQ7\|VMA21_HUMAN | Vacuolar ATPase assembly integral membrane protein VMA21 | 101 | 11354 | 1.68 | 11.87999994 |
| sp\|P51636\|CAV2_HUMAN | Caveolin-2 | 162 | 18291 | 2 | 9.877000004 |
| sp\|Q15907\|RB11B_HUMAN | Ras-related protein Rab-11B | 218 | 24488.3 | 8.43 | 26.1500001 |
| sp\|P62879\|GBB2_HUMAN | Guanine nucleotide-binding protein G(I)/G(S)/G(T) subunit beta-2 | 340 | 37330.6 | 2 | 5.587999895 |
| sp\|P62906\|RL10A_HUMAN | 60S ribosomal protein L10a | 217 | 24831.1 | 7.06 | 17.04999954 |
| sp\|Q12907\|LMAN2_HUMAN | Vesicular integral-membrane protein VIP36 | 356 | 40228.4 | 3.37 | 6.460999697 |
| sp\|O00299\|CLIC1_HUMAN | Chloride intracellular channel protein 1 | 241 | 26922.5 | 23.91 | 53.93999815 |
| sp\|Q13151\|ROA0_HUMAN | Heterogeneous nuclear ribonucleoprotein A0 | 305 | 30840.6 | 1.72 | 12.46000007 |
| sp\|P46776\|RL27A_HUMAN | 60S ribosomal protein L27a | 148 | 16561.4 | 3.77 | 8.78399983 |
| sp\|P62136\|PP1A_HUMAN | Serine/threonine-protein phosphatase PP1-alpha catalytic subunit | 330 | 37511.7 | 3.4 | 9.696999937 |
| sp\|O14828\|SCAM3_HUMAN | Secretory carrier-associated membrane protein 3 | 347 | 38286.6 | 2 | 4.611000046 |
| sp\|P0DOX8\|IGL1_HUMAN | Immunoglobulin lambda-1 light chain | 216 | 22830 | 2.25 | 16.67000055 |
| sp\|P62081\|RS7_HUMAN | 40S ribosomal protein S7 | 194 | 22126.7 | 7.38 | 18.55999976 |
| sp\|P16152\|CBR1_HUMAN | Carbonyl reductase [NADPH] 1 | 277 | 30374.7 | 3.14 | 9.747000039 |
| sp\|P13804\|ETFA_HUMAN | Electron transfer flavoprotein subunit alpha, mitochondrial | 333 | 35079.2 | 2.03 | 3.903999925 |
| sp\|P05388\|RLA0_HUMAN | 60S acidic ribosomal protein P0 | 317 | 34273.2 | 13.03 | 30.59999943 |
| sp\|Q9H3N1\|TMX1_HUMAN | Thioredoxin-related transmembrane protein 1 | 280 | 31790.9 | 1.45 | 4.286000133 |
| sp\|P49721\|PSB2_HUMAN | Proteasome subunit beta type-2 | 201 | 22836 | 3.51 | 12.94 |
| sp\|E9PAV3\|NACAM_HUMAN | Nascent polypeptide-associated complex subunit alpha, muscle-specific form | 2078 | 205419 | 4 | 1.396000013 |
| sp\|Q9UBQ5\|EIF3K_HUMAN | Eukaryotic translation initiation factor 3 subunit K | 218 | 25059.4 | 5.14 | 13.30000013 |
| sp\|P00492\|HPRT_HUMAN | Hypoxanthine-guanine phosphoribosyltransferase | 218 | 24579.2 | 3.54 | 11.4699997 |
| sp\|P62805\|H4_HUMAN | Histone H4 | 103 | 11367.3 | 6.94 | 33.98000002 |
| sp\|Q13347\|EIF3I_HUMAN | Eukaryotic translation initiation factor 3 subunit I | 325 | 36501.6 | 4.58 | 12.62000054 |
| sp\|P62750\|RL23A_HUMAN | 60S ribosomal protein L23a | 156 | 17694.9 | 5.65 | 16.03000015 |
| sp\|Q9NP72\|RAB18_HUMAN | Ras-related protein Rab-18 | 206 | 22976.9 | 2.41 | 5.339999869 |
| sp\|P09382\|LEG1_HUMAN | Galectin-1 | 135 | 14715.6 | 1.91 | 20.73999941 |
| sp\|Q99714\|HCD2_HUMAN | 3-hydroxyacyl-CoA dehydrogenase type-2 | 261 | 26922.9 | 11.46 | 40.61000049 |
| sp\|P52565\|GDIR1_HUMAN | Rho GDP-dissociation inhibitor 1 | 204 | 23206.9 | 6.76 | 31.8599999 |
| sp\|P30084\|ECHM_HUMAN | Enoyl-CoA hydratase, mitochondrial | 290 | 31387.1 | 7.24 | 22.40999937 |
| sp\|P30044\|PRDX5_HUMAN | Peroxiredoxin-5, mitochondrial | 214 | 22086.2 | 6.12 | 23.36000055 |
| sp\|Q99497\|PARK7_HUMAN | Protein/nucleic acid deglycase DJ-1 | 189 | 19890.9 | 6.39 | 23.81000072 |
| sp\|Q99426\|TBCB_HUMAN | Tubulin-folding cofactor B | 244 | 27325.3 | 2.82 | 4.098000005 |
| sp\|P62266\|RS23_HUMAN | 40S ribosomal protein S23 | 143 | 15807.5 | 3.41 | 15.37999958 |
| sp\|Q99729\|ROAA_HUMAN | Heterogeneous nuclear ribonucleoprotein A/B | 332 | 36224.8 | 3.96 | 6.927999854 |
| sp\|P25789\|PSA4_HUMAN | Proteasome subunit alpha type-4 | 261 | 29483.6 | 7.95 | 22.99000025 |
| sp\|P83731\|RL24_HUMAN | 60S ribosomal protein L24 | 157 | 17778.8 | 3.74 | 12.73999959 |
| sp\|Q15365\|PCBP1_HUMAN | Poly(rC)-binding protein 1 | 356 | 37497.5 | 7.7 | 15.17000049 |
| sp\|P25398\|RS12_HUMAN | 40S ribosomal protein S12 | 132 | 14514.8 | 3.47 | 33.32999945 |
| sp\|P18085\|ARF4_HUMAN | ADP-ribosylation factor 4 | 180 | 20510.6 | 3.91 | 17.21999943 |
| sp\|P08865\|RSSA_HUMAN | 40S ribosomal protein SA | 295 | 32853.8 | 2.27 | 10.17000005 |
| sp\|P06748\|NPM_HUMAN | Nucleophosmin | 294 | 32574.8 | 5.49 | 16.67000055 |
| sp\|Q06830\|PRDX1_HUMAN | Peroxiredoxin-1 | 199 | 22110.2 | 18.87 | 44.72000003 |
| sp\|P09651\|ROA1_HUMAN | Heterogeneous nuclear ribonucleoprotein A1 | 372 | 38746.7 | 6.78 | 12.63000071 |
| sp\|P29692\|EF1D_HUMAN | Elongation factor 1-delta | 281 | 31121.6 | 8.6 | 23.83999974 |
| sp\|P14618\|KPYM_HUMAN | Pyruvate kinase PKM | 531 | 57936.4 | 4.88 | 8.663000166 |
| sp\|P35080\|PROF2_HUMAN | Profilin-2 | 140 | 15046.2 | 2 | 10.00000015 |
| sp\|P16949\|STMN1_HUMAN | Stathmin | 149 | 17302.4 | 3.05 | 15.44000059 |
| sp\|O75323\|NIPS2_HUMAN | Protein NipSnap homolog 2 | 286 | 33742.4 | 1.8 | 3.147000074 |
| sp\|P31942\|HNRH3_HUMAN | Heterogeneous nuclear ribonucleoprotein H3 | 346 | 36926.4 | 2 | 3.468000144 |
| sp\|Q00325\|MPCP_HUMAN | Phosphate carrier protein, mitochondrial | 362 | 40094.5 | 2.75 | 5.248999968 |
| sp\|P67936\|TPM4_HUMAN | Tropomyosin alpha-4 chain | 248 | 28521.5 | 4.78 | 24.1899997 |
| sp\|Q13011\|ECH1_HUMAN | Delta(3,5)-Delta(2,4)-dienoyl-CoA isomerase, mitochondrial | 328 | 35815.8 | 13.48 | 22.56000042 |
| sp\|P08574\|CY1_HUMAN | Cytochrome c1, heme protein, mitochondrial | 325 | 35421.6 | 1.3 | 4.922999814 |
| sp\|Q14847\|LASP1_HUMAN | LIM and SH3 domain protein 1 | 261 | 29717.1 | 3.89 | 9.578999877 |
| sp\|P31949\|S10AB_HUMAN | Protein S100-A11 | 105 | 11740.3 | 2.27 | 9.523999691 |
| sp\|P26641\|EF1G_HUMAN | Elongation factor 1-gamma | 437 | 50118.4 | 2.58 | 2.975000069 |
| sp\|P32969\|RL9_HUMAN | 60S ribosomal protein L9 | 192 | 21863.3 | 13.06 | 32.28999972 |
| sp\|Q86V81\|THOC4_HUMAN | THO complex subunit 4 | 257 | 26887.7 | 2.04 | 7.004000247 |
| sp\|P50914\|RL14_HUMAN | 60S ribosomal protein L14 | 215 | 23431.7 | 6.38 | 23.72000068 |
| sp\|P60981\|DEST_HUMAN | Destrin | 165 | 18505.5 | 2.43 | 6.667000055 |
| sp\|Q16563\|SYPL1_HUMAN | Synaptophysin-like protein 1 | 259 | 28565 | 1.66 | 4.247000068 |
| sp\|Q9NVJ2\|ARL8B_HUMAN | ADP-ribosylation factor-like protein 8B | 186 | 21538.8 | 2.61 | 12.37000003 |
| sp\|O75396\|SC22B_HUMAN | Vesicle-trafficking protein SEC22b | 215 | 24593.1 | 2.06 | 6.511999667 |
| sp\|P51149\|RAB7A_HUMAN | Ras-related protein Rab-7a | 207 | 23489.6 | 14.78 | 39.61000144 |
| sp\|P05141\|ADT2_HUMAN | ADP/ATP translocase 2 | 298 | 32852 | 23.65 | 32.89000094 |
| sp\|O95336\|6PGL_HUMAN | 6-phosphogluconolactonase | 258 | 27546.5 | 2.07 | 6.202000007 |
| sp\|P61604\|CH10_HUMAN | 10 kDa heat shock protein, mitochondrial | 102 | 10931.6 | 4.25 | 23.53000045 |
| sp\|P04075\|ALDOA_HUMAN | Fructose-bisphosphate aldolase A | 364 | 39419.7 | 17.6 | 32.69000053 |
| sp\|P36542\|ATPG_HUMAN | ATP synthase subunit gamma, mitochondrial | 298 | 32995.7 | 2.07 | 4.02700007 |
| sp\|P61081\|UBC12_HUMAN | NEDD8-conjugating enzyme Ubc12 | 183 | 20899.8 | 3.05 | 14.74999934 |
| sp\|P61247\|RS3A_HUMAN | 40S ribosomal protein S3a | 264 | 29944.8 | 20.96 | 37.5 |
| sp\|Q07020\|RL18_HUMAN | 60S ribosomal protein L18 | 188 | 21634.3 | 9.29 | 26.06000006 |
| sp\|P50897\|PPT1_HUMAN | Palmitoyl-protein thioesterase 1 | 306 | 34193.2 | 1.93 | 4.90199998 |
| sp\|P30050\|RL12_HUMAN | 60S ribosomal protein L12 | 165 | 17818.4 | 9.18 | 33.32999945 |
| sp\|P37108\|SRP14_HUMAN | Signal recognition particle 14 kDa protein | 136 | 14569.8 | 3.07 | 17.64999926 |
| sp\|O00151\|PDLI1_HUMAN | PDZ and LIM domain protein 1 | 329 | 36071.5 | 1.72 | 4.558999836 |
| sp\|Q9H8S9\|MOB1A_HUMAN | MOB kinase activator 1A | 216 | 25079.6 | 3.19 | 10.64999998 |
| sp\|Q13162\|PRDX4_HUMAN | Peroxiredoxin-4 | 271 | 30539.6 | 2.14 | 11.06999964 |
| sp\|P62280\|RS11_HUMAN | 40S ribosomal protein S11 | 158 | 18430.6 | 13.2 | 44.94000077 |
| sp\|O00170\|AIP_HUMAN | AH receptor-interacting protein | 330 | 37635.7 | 2 | 4.241999984 |
| sp\|P52907\|CAZA1_HUMAN | F-actin-capping protein subunit alpha-1 | 286 | 32922.5 | 9.63 | 26.21999979 |
| sp\|O75822\|EIF3J_HUMAN | Eukaryotic translation initiation factor 3 subunit J | 258 | 29062.2 | 2.02 | 4.650999978 |
| sp\|Q02543\|RL18A_HUMAN | 60S ribosomal protein L18a | 176 | 20762.2 | 6.2 | 17.04999954 |
| sp\|P22392\|NDKB_HUMAN | Nucleoside diphosphate kinase B | 152 | 17297.9 | 9.43 | 44.08000112 |
| sp\|P04406\|G3P_HUMAN | Glyceraldehyde-3-phosphate dehydrogenase | 335 | 36053 | 35.31 | 60.90000272 |


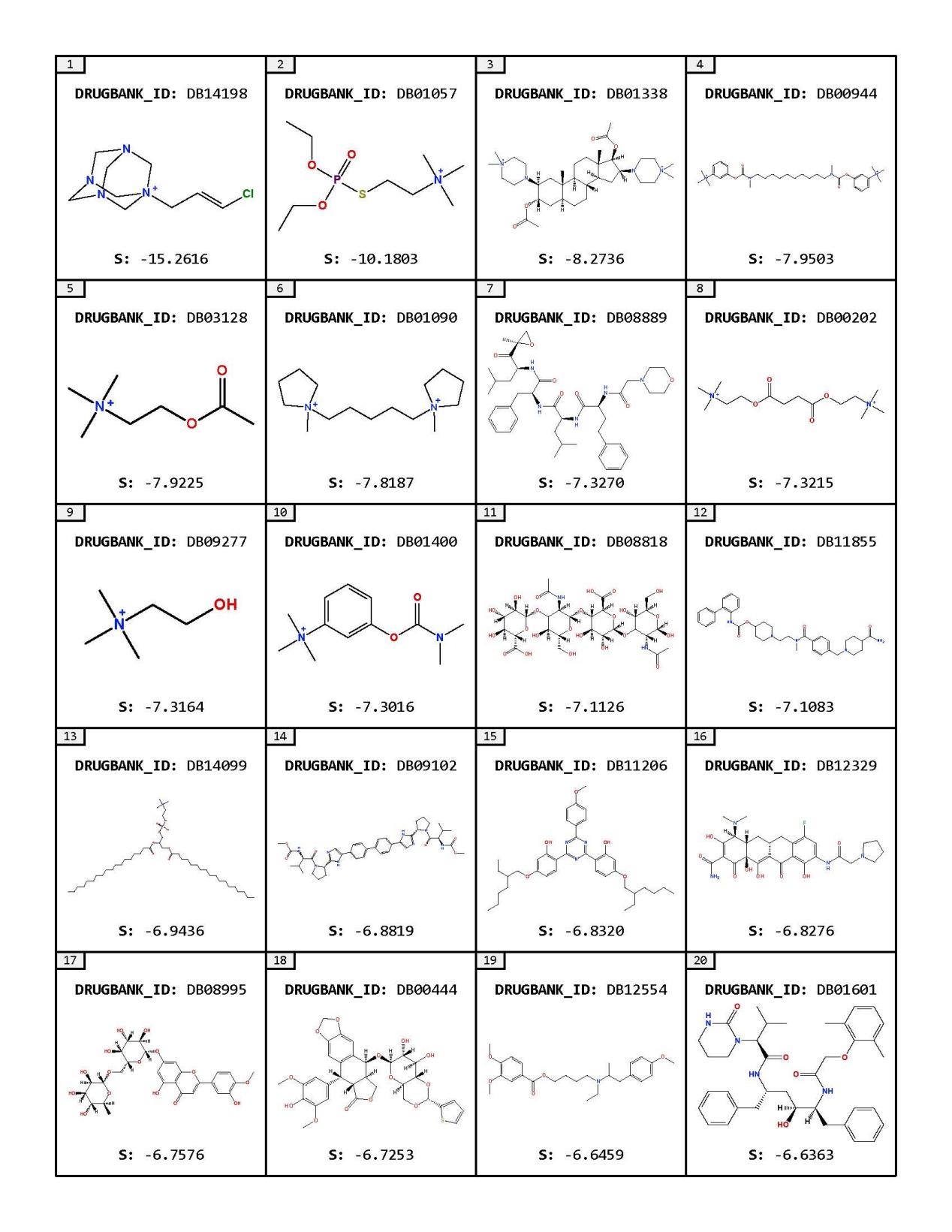


Supplementary Figure S1. Structures and docking scores of the top 20 inhibitors of the HTRA1.


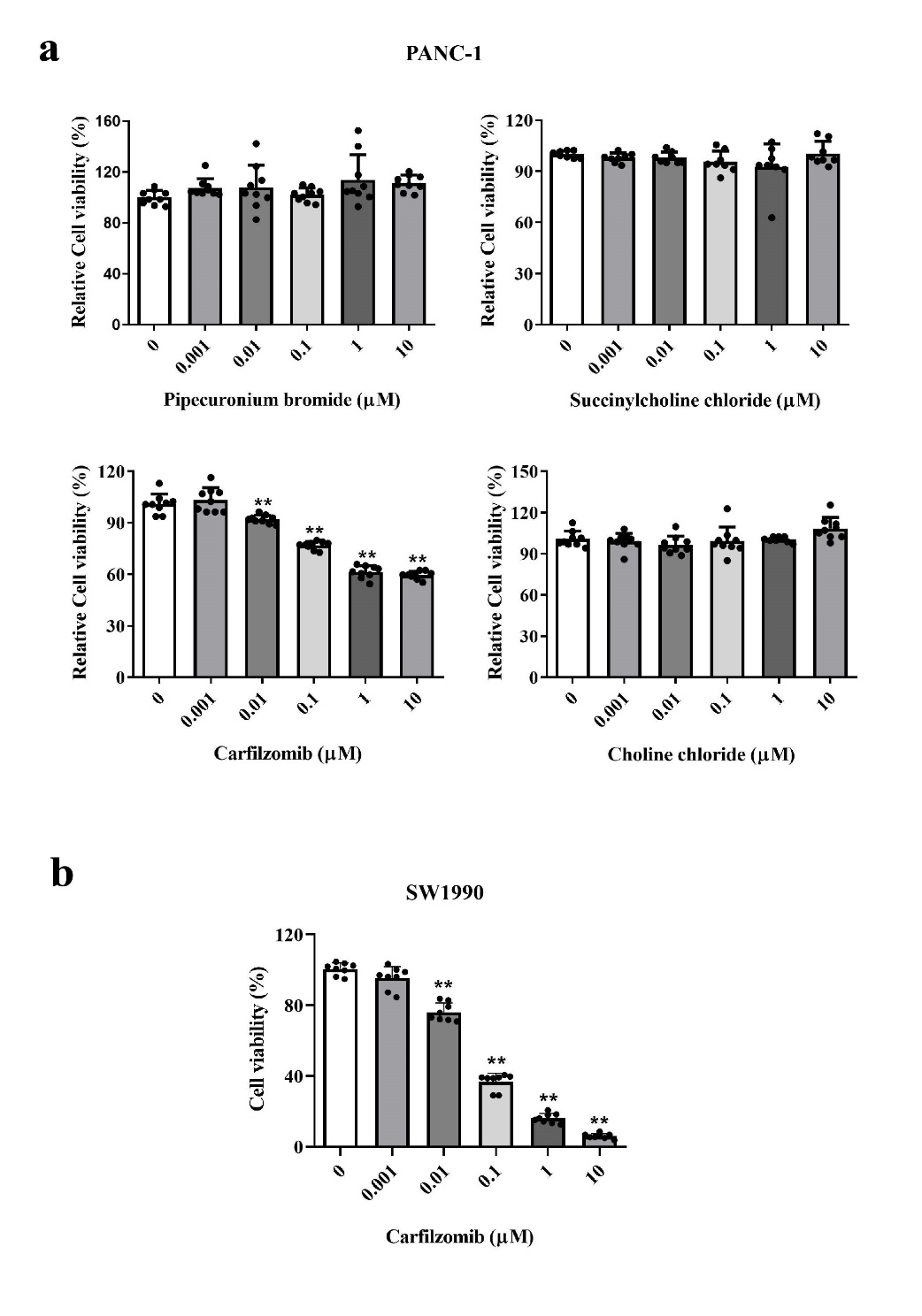


Supplementary Figure S2. (a) Toxicities of the selected 4 compounds in PANC-1 in vitro (n = 9). (b) Toxicities of carfilzomib inSW1990 in vitro (n = 8).
